# Trajectory planning method for pipeline installation robots based on AS-DTRRT

**DOI:** 10.1371/journal.pone.0346076

**Published:** 2026-05-22

**Authors:** Zhixiang Liu, Wentao Hu, Chunxue Xie

**Affiliations:** 1 Institute of Mineral Resources, Liaoning Technical University, Fuxin, Liaoning, China; 2 School of Mechanical Engineering, Liaoning Technical University, Fuxin, Liaoning, China; 3 School of Mechanics & Engineering, Liaoning Technical University, Fuxin, Liaoning, China; Beijing Institute of Technology, CHINA

## Abstract

Pipeline installation robots install pipelines on both sides of mine roadways using robotic arms; the geometric shape and length of the pipelines affect the movement trajectory of the robotic arms, and the working environment is complex and changeable. Aiming at the issues of the mechanical arm’s long motion trajectory, low installation efficiency, and pipeline collision vulnerability during installation, a trajectory planning method based on the improved dual-tree RRT algorithm (AS-DTRRT) is proposed. Taking the pipeline installation robot with a 6-degree-of-freedom Cartesian coordinate robotic arm as the research object, the Artificial Potential Field method is introduced to guide the growth direction of new nodes in the path of the dual-tree RRT algorithm; aiming at the problem that the Artificial Potential Field method is prone to falling into local optimality, an adaptive step-size dynamic environment bias strategy is proposed, which automatically adjusts the step size according to the environmental conditions of the position where the new node is located to further improve the path search capability. This study analyzes the influence of pipeline geometry and length on the robotic arm’s working space, proposes a pipeline collision avoidance strategy considering the working environment, and applies quintic polynomial interpolation to plan the trajectory of collision-free paths while considering joint speed and acceleration constraints. Simulations and platform experiments comparing AS-DTRRT with RRT*, D-RRT, Bi-RRT*, and A-RRT validate the method’s feasibility, demonstrating that AS-DTRRT outperforms the four algorithms by reducing actual runtime by 20.22%, 15.48%, 4.69%, and 16.47% and shortening average path lengths by 21.17%, 13.78%, 6.85%, and 9.24% respectively. The proposed method enhances search efficiency and reduces path lengths, providing an effective approach for trajectory planning of pipeline installation robots.

## 1. Introduction

Coal serves as the energy foundation of the country and a prerequisite for promoting the high-quality development of China’s economy [1]. Therefore, the coal industry must undergo intelligent upgrading of coal mines to enhance operational efficiency [2,3]. The stable operation of underground systems such as ventilation, drainage, and gas ex-traction highly relies on the efficient laying and maintenance of pipeline networks, with the quality of pipeline installation directly impacting mine safety production and resource extraction efficiency [4,5]. Pipeline installation robots are vital equipment in coal mine operations. These robots use mechanical arms to install pipelines on both sides of mine tunnels, where the pose of the mechanical arm holding the pipeline changes with the pipeline’s position. Given the complex and changeable working environment, the trajectory of the mechanical arm is of critical importance [6]. Pipelines are generally long, making them prone to collisions with surrounding environmental obstacles. During operation, pipeline installation robots must not only ensure obstacle avoidance for their mechanical arms [7] but also for the pipelines clamped by mechanical claws. The installation of long-distance pipelines remains a persistent challenge in the industry. Existing pipeline installation robots struggle with autonomous trajectory planning in confined and restricted environments. Although trajectory planning technologies have made progress, achieving a balance between real-time computational efficiency and kinematic feasibility analysis in unstructured narrow spaces remains difficult. Therefore, developing pipeline installation robots capable of autonomous planning and obstacle avoidance in complex environments is crucial for advancing intelligent transformation in the coal mining sector.

The latest research advancements in autonomous trajectory planning technology are as follows: Regarding aerial systems, Eskandari et al. [7] proposed a Visual GAN (VGAN) framework for end-to-end UAV trajectory generation in RIS-assisted networks, which processes bird’s eye view images to optimize energy-efficient communication and navigation feasibility. In terms of ground vehicle intelligence, Chen et al. [8] developed a personalized longitudinal motion planning policy by synergistically combining reinforcement learning with imitation learning to align autonomous driving styles with human driver characteristics. Furthermore, to address complex urban traffic, Chi et al. [9,10] introduced a spatiotemporal-restricted A algorithm* to support lane-free navigation at intersections with mixed flows, utilizing multiple spatiotemporal constraints to accelerate solution efficiency and improve traffic flow. These diverse methodologies collectively demonstrate the shift toward more adaptive and intelligent planning frameworks.

In the field of robot trajectory planning, scholars have conducted extensive research. For example, Cao Xiangang et al. [11] proposed an environment-sensitive target biasing strategy, which adjusts the target biasing probability threshold in real-time according to environmental changes, alters the randomness of new node expansion, and reduces the probability of local optima. Yao Yafeng et al. [12] developed a three-stage obstacle avoidance trajectory planning strategy. Zhang Daiyu et al. [13], Li Z et al. [14], and Liu Yuwei et al. [15] proposed the Ant-BiRRT algorithm, a bidirectional rapidly exploring random tree with node culling based on ant colony optimization. Bian Z et al. [16] and Li Yong [17] introduced the improved APF-DDPG algorithm, combining the artificial potential field method (APF) and deep deterministic policy gradient algorithm (DDPG). Improved RRT [18] and the RRT-Connect algorithm [19] are also widely applied in trajectory planning. Wu Song et al. [20] proposed a hybrid RRT algorithm, improving directivity by adjusting node derivation directions while shortening the planned path. Liang et al. [21] proposed an adaptive biasing probability sampling strategy to dynamically adjust the target bias threshold, optimizing the selection of random sampling points and new node generation directions to reduce the search space and enhance efficiency [22]. Zhang Chao et al. [23] developed a trajectory planning method based on the improved NSGA-II. Zhang Kekun et al. [24] proposed an autonomous path planning method for underground handling robots using improved A* and DWA algorithms. Wang Hongwei et al. [25–27] proposed path planning combining improved A* and potential field methods to enhance search efficiency. Gao Jiacai et al. [28] introduced a two-level collision detection algorithm for mechanical arms and obstacles. Gao Feixiang et al. [29] studied obstacle avoidance path planning using improved artificial potential field methods, improving obstacle avoidance efficiency.

The aforementioned deep learning methods and traditional improved RRT algorithms have enhanced trajectory planning autonomy and improved path quality with higher search efficiency. However, challenges persist in narrow passages and confined tunnels. Deep learning techniques heavily rely on large-scale annotated data or high-precision simulators, resources that are often unavailable in specific coal mining environments. Traditional RRT algorithms exhibit reduced probability of node exploration in narrow gaps, leading to search stagnation or excessive iterations. For long-distance pipeline grasping tasks requiring extensive search space coverage, the lack of guidance mechanisms in RRT algorithms results in excessively long trajectories or search failures.

Aiming at the issues of low search efficiency, long path length, and pipeline collisions, this paper proposes a trajectory planning method based on the improved dual-tree RRT algorithm (AS-DTRRT). Compared with other existing RRT-based improvements, the AS-DTRRT search algorithm is proposed for the first time, which combines the advantages of double-tree RRT and artificial potential field method such as speed and target guidance, and introduces an adaptive step size environment bias strategy, which solves the problems of easy to fall into the optimal solution when node expansion, generates a large number of invalid nodes and shortest paths, and improves the search efficiency. The improved dual-tree RRT algorithm is used for path planning to obtain the shortest collision-free path for pipeline installation robots and extract key motion sequences [30–33]. Task constraints between pipelines and the environment are established to adjust path key points, ensuring pipeline postures and enabling obstacle avoidance [33–34]. Quintic polynomial interpolation is applied to the planned path for trajectory smoothing, reducing operational impact. Experimental results verify that the planned trajectory meets pipeline obstacle avoidance requirements, with both planning time and path length significantly reduced. This study provides technical support for trajectory planning of similar coal mine robots.

## 2. Related works

### 2.1. Composition of pipeline installation robot

**Fig 1:**
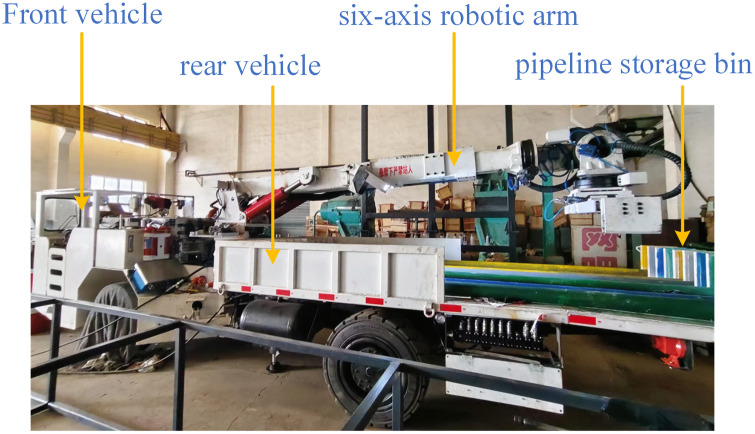
Overall structure of pipeline installation robot.

The pipeline installation robot is generally composed of a vehicle body structure and a mechanical arm structure, as shown in Fig 1. The vehicle body structure mainly includes: front vehicle, rear vehicle, and pipeline bin. The front vehicle is mainly responsible for vehicle driving, while the rear vehicle is mainly responsible for loading pipe-lines and carrying the mechanical arm. The vehicle body structure adopts an articulated chassis, which can realize the integration of various working mechanisms and flexible movement in mines, including small-range pose adjustment and medium-short distance movement. The mechanical arm is mainly used for the installation of pipelines of different specifications.

### 2.2. Structural analysis of mechanical arm for pipeline installation robot

**Fig 2:**
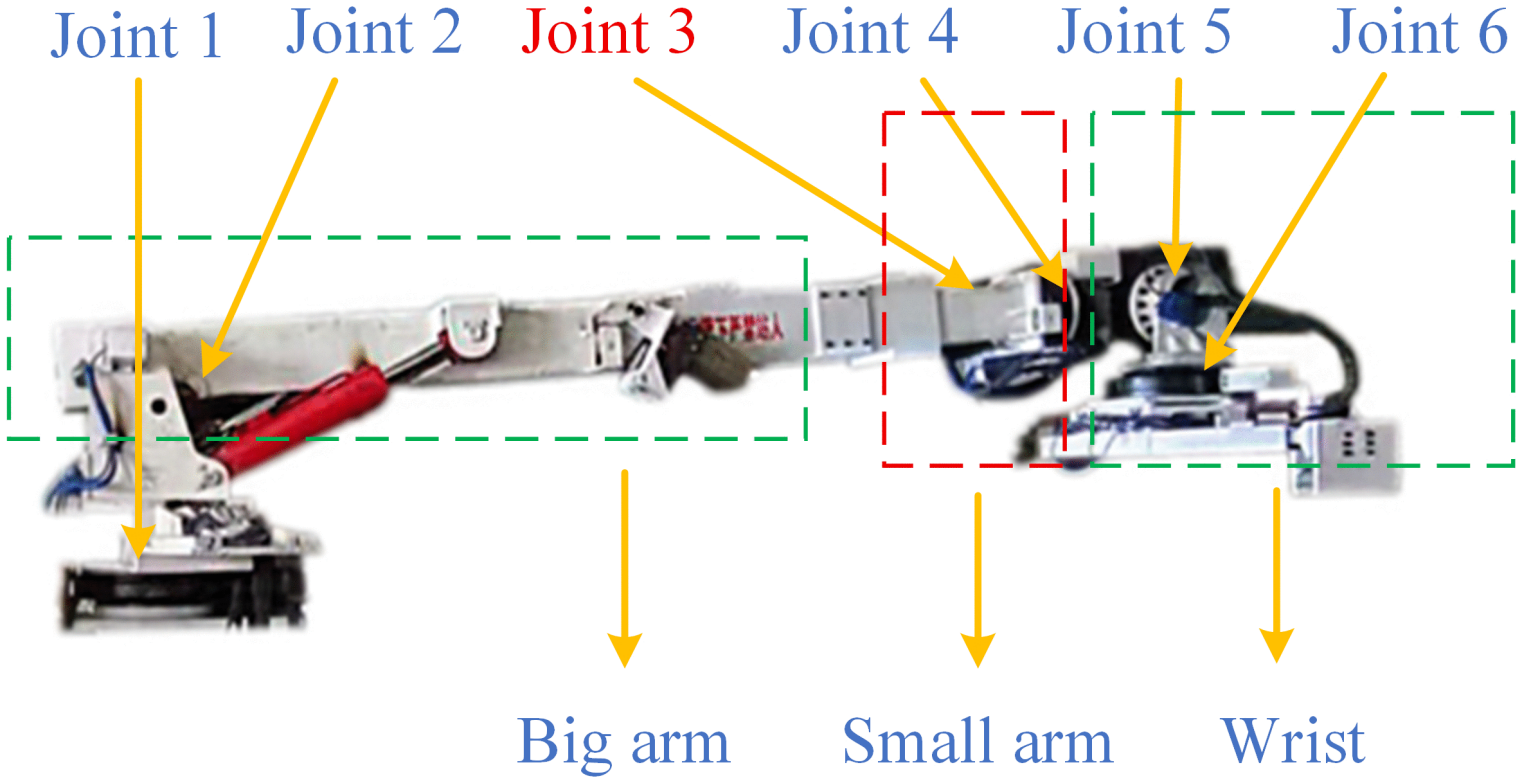
Robotic arm structure of pipeline installation robot.

The robotic arm structure of the pipeline installation robot is shown in Fig 2, which has six axes in total. Among them, joint 3 is a prismatic joint, and the rest are revolute joints. Joint 1 is used for the extension and retraction of the big arm, joint 2 for the pitching of the big arm, joint 3 for the extension and retraction of the small arm, and joints 4, 5 and 6 for the rotation of the wrist.

### 2.3. Smart pipeline lifting and gripping control system scheme

**Fig 3:**
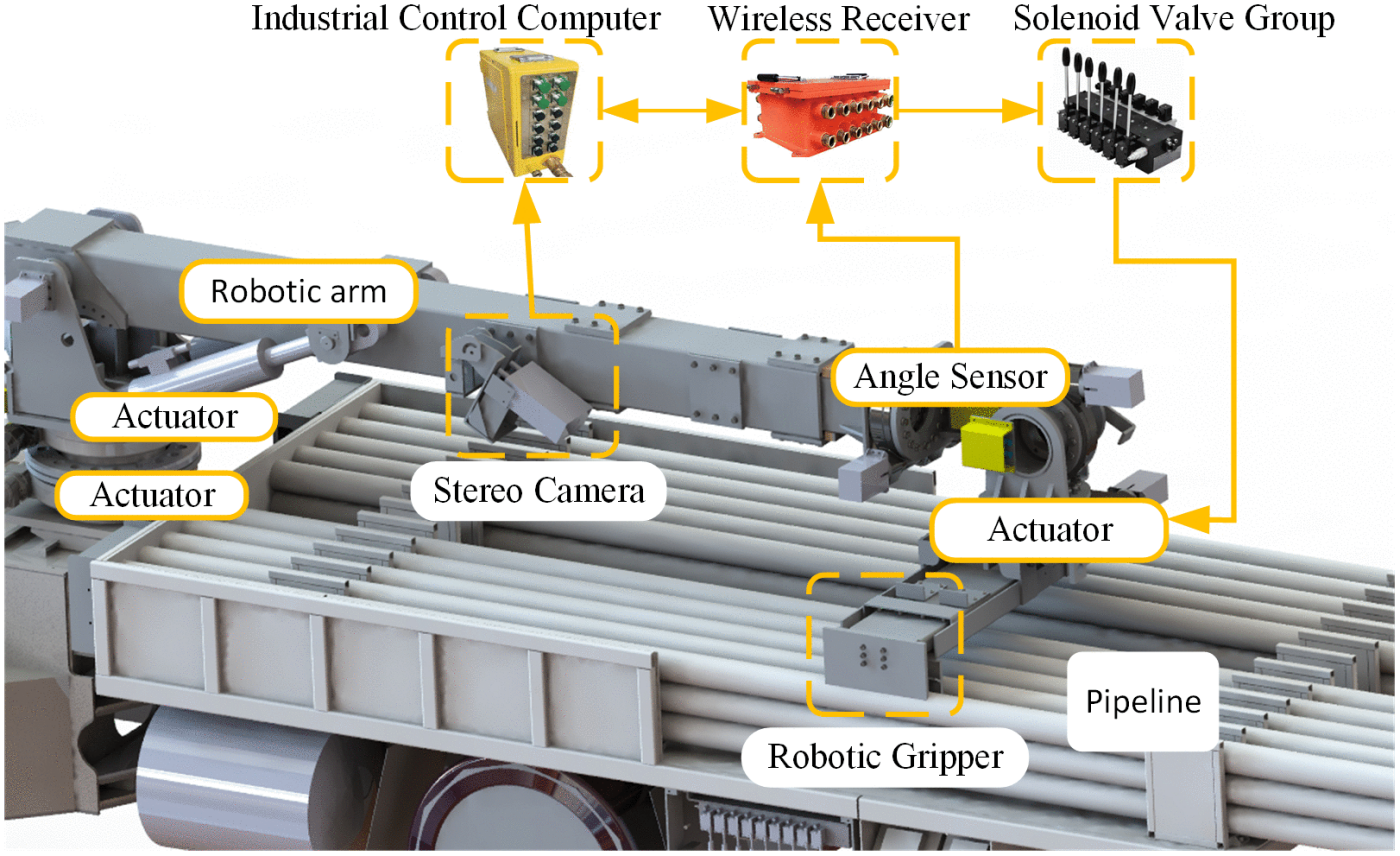
Intelligent pipeline grasping control system.

This study intends to use a binocular vision camera for pipeline color and position recognition, and then control the robotic arm to complete autonomous grabbing and placing of pipelines. The hardware of the intelligent pipeline grabbing control system mainly includes a binocular camera for pipeline color and position recognition, robotic arm joint angle detection sensors, an industrial computer for algorithm processing, a controller for controlling robotic arm movement, and a solenoid valve group, etc. The system scheme is shown in the Fig 3:

The control system mainly consists of two parts:

(1) Sensing and Detection Unit:

The motion posture detection of the robotic arm employs five mine-used intrinsically safe encoders (BQH12) and one mine-used intrinsically safe dis-placement sensor (GUC2000). The camera adopted is a mine-used intrinsically safe camera (KBA12).

(2) Control Unit:

The industrial computer (IPC610) and the mine-used flameproof and intrinsically safe vehicle mounted wireless receiver (FWS100C)

(3) Actuation Mechanism:

The valve group controlling the robotic arm movement adopts a mine-used intrinsically safe solenoid valve group (STPSV55C2C/240–3).

### 2.4. Forward kinematic modeling of robotic arm

**Fig 4:**
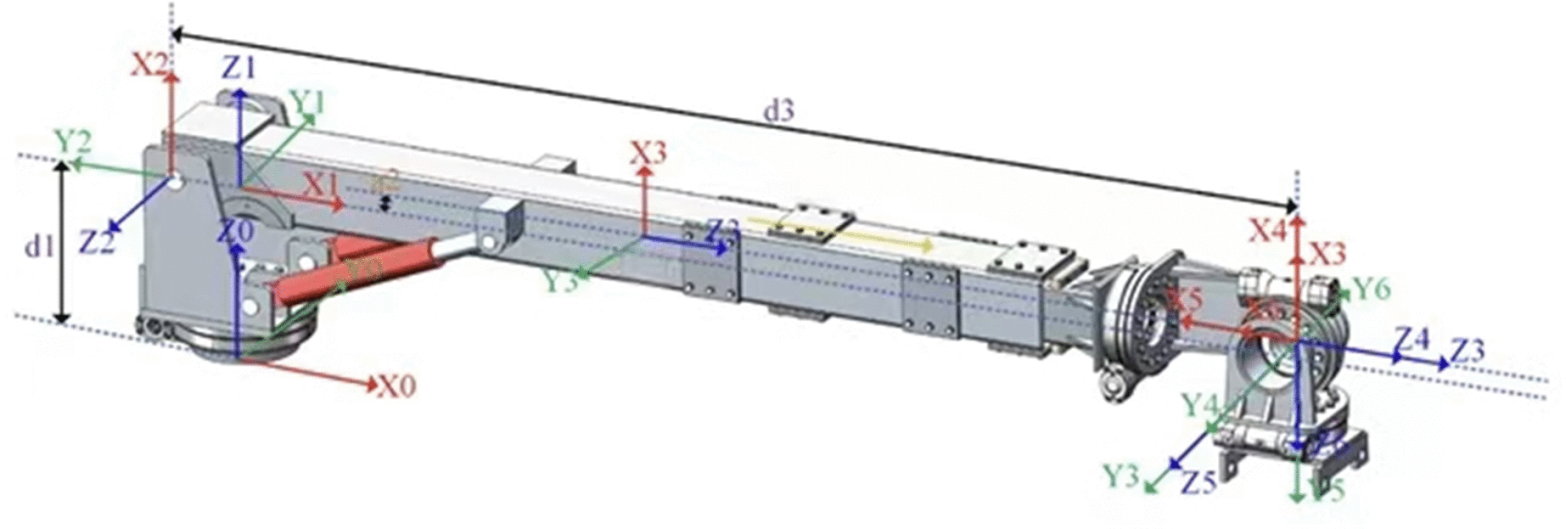
D-H model of the robotic arm of the pipeline installation robot.

This study employs an improved Denavit-Hartenberg (D-H) parameter method to establish the kinematic model of the pipeline installation robot’s robotic arm. The specific implementation process is as follows: The six link components of the robotic arm are sequentially coded as 0–6 from the base component to the end-effector. In accordance with modeling specifications, the base coordinate system {0} is set at the joint of the robotic arm slewing bearing mechanism and the body. A complete kinematic analysis framework is formed by systematically constructing coordinate systems for each link. This method accurately describes the pose transformation relationship between adjacent links through four-dimensional parameters (joint angle θ, link offset d, link length a, link twist angle α). The specific parameter configurations are de-tailed in Table 1, and the D-H model of the pipeline installation robot’s robotic arm is shown in Fig 4.

**Table 1:** Table of D - H parameters of the robotic arm.

Link i	$a_{i-1}/$ °	$a_{i-1}/mm$	$d_{i}/mm$	$\theta_{i}/$ °
1	0	0	$d_{\text{1}}$	$\theta_{\text{1}}$
2	90°	$a_{\text{1}}$	0	$\theta_{\text{2}}$
3	90°	$a_{\text{2}}$	$d_{\text{3}}$	$\theta_{\text{3}}$
4	0	0	0	$\theta_{\text{4}}$
5	−90°	0	0	$\theta_{\text{5}}$
6	−90°	0	$d_{\text{6}}$	$\theta_{\text{6}}$

**Fig 5:**
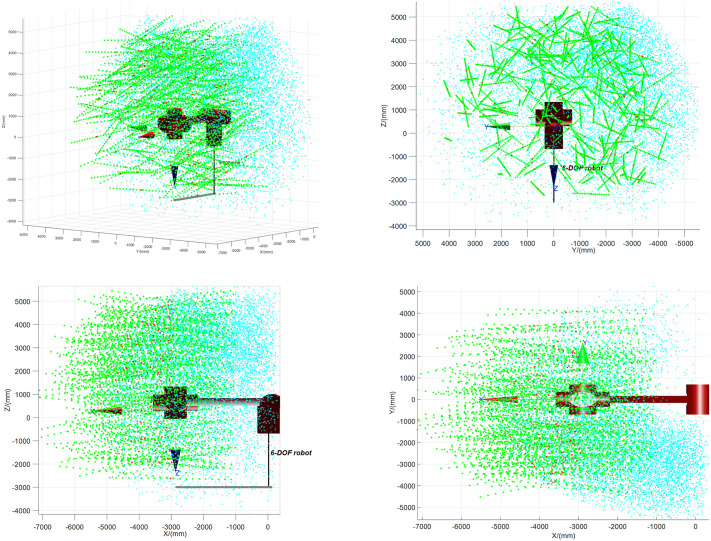
The workspace of robotic arm after grabbing a pipeline. **(a)** X-Y-Z plane workspace; **(b)** Y-Z plane workspace; **(c)** X-Z plane workspace; **(d)** X-Y plane workspace.

In the parameters, a_2_ = 25, d_1_ = 659, d_3_ = 3833, and d_6_ = 35. Based on the DH parameter table, a robotic arm model was established in MATLAB. With the joint angle limits of the six axes, the Monte Carlo method was used to randomly sample 10,000 working points at the robotic arm’s end to calculate the workspace, as shown in Fig 5. The cyan point cloud represents the original end workspace. After the robotic arm grasps the pipeline, the pipeline’s length and angle restrict the robotic arm’s movement. With pipeline constraints added, the workspace of the robotic arm after grasping the pipeline is the red point cloud in Fig 5, where the green line denotes the pipeline’s central axis.

### 2.5. Inverse kinematics of mechanical arm

This study employs matrix transformation techniques in algebraic analysis for inverse kinematics solution. The specific implementation process is as follows: By left-multiplying the transformation equation with inverse matrices, the complex pose matrix is gradually decomposed into trigonometric function equations solvable independently, and joint parameters are sequentially calculated using the variable separation method. This systematic solution strategy ensures calculation accuracy while effectively avoiding the computational redundancy of traditional geometric methods in multi-degree-of-freedom systems.


$$\begin{array}{c} \theta_{\text{1}}=arctan2\left(p_{x},p_{y}\right) \end{array}$$ (1)

$$\begin{array}{c} \theta_{\text{2}}\;=arctan2\left(B,A\right)\pm arccos\left(\;\frac{C}{\sqrt{A_{\text{2}}+B_{\text{2}}}}\;\right) \end{array}$$ (2)

$$\begin{array}{c} d_{\text{3}}=\frac{K-a_{\text{1}}-a_{\text{2}}c_{\text{2}}}{s_{\text{2}}} \end{array}$$ (3)

$$\begin{array}{c} \theta_{\text{4}}=arctan\left(\frac{a_{x}s_{\text{1}}-a_{y}c_{\text{1}}}{a_{z}s_{\text{2}}+a_{x}c_{\text{1}}c_{\text{2}}+a_{y}c_{\text{2}}s_{\text{1}}}\right) \end{array}$$ (4)

$$\begin{array}{c} \theta_{\text{5}}=\pm arccos\left(a_{z}c_{\text{2}}-a_{x}c_{\text{1}}s_{\text{2}}-a_{y}s_{\text{1}}s_{\text{2}}\right) \end{array}$$ (5)

$$\begin{array}{c} \theta_{\text{6}}=arctan\left(\frac{o_{x}c_{\text{1}}s_{\text{2}}-o_{z}c_{\text{2}}+o_{y}s_{\text{1}}s_{\text{2}}}{-n_{x}c_{\text{1}}s_{\text{2}}+n_{z}c_{\text{2}}-n_{y}s_{\text{1}}s_{\text{2}}}\right) \end{array}$$ (6)

## 3. Methodology

### 3.1. Path search algorithms

#### 3.1.1. Improved Dual-Tree RRT.

The dual-tree RRT algorithm constructs two trees simultaneously from the start state and the target state, performing random sampling and expanding toward the sampling points until the two trees are connected. The artificial potential field method is a path planning approach that guides the robot to move toward the target and avoid obstacles by constructing attractive and repulsive potential fields.

**Fig 6:**
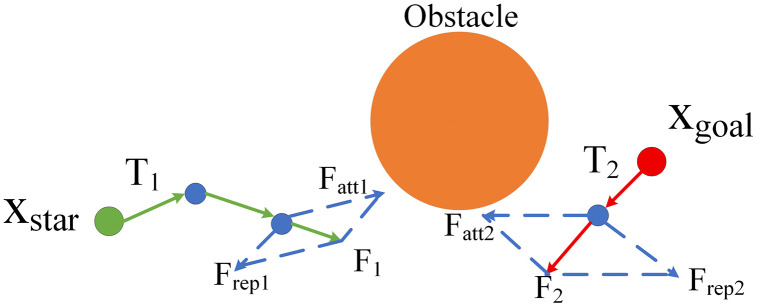
Introduction of artificial potential field in double-tree RRT.

The dual-tree RRT exhibits arbitrariness and blindness when generating new nodes, leading to numerous invalid paths during tree growth and reducing search efficiency. Therefore, the artificial potential field method is introduced. As shown in Fig 6, X_star_ is the starting point, X_goal_ is the target point, and Obstacle denotes the obstacle. T_1_ and T_2_ are the generated and retained paths, F_att1_ and F_att2_ are attractive forces, F_rep1_ and F_rep2_ are repulsive forces, and F_1_ and F_2_ are the resultant forces. The resultant force di-rection generated by the artificial potential field method is utilized to guide the generation of new nodes, thereby directing the rapid connection of the dual trees, reducing the generation of redundant nodes, and improving search efficiency.

Gravitational Field Function:


$$\begin{array}{c} U_{att}(x)=\;\frac{\text{1}}{\text{2}}\varphi_{att}\;{\left[d(x,x_{goal})\right]}^{\text{2}} \end{array}$$ (7)

$\varphi_{att}$ is the gravitational coefficient, x is the position of an arbitrary node, $x_{goal}$ is the position of the target point, and $d\left(x,x_{goal}\right)$ is the distance between the node and the target point. The direction of the gravitational force is from the node to the target point, attracting the node to approach the target point.

Repulsive Field Function:


$$\begin{array}{c} U_{rep}\left(x\right)\text{\textbackslash{}hspace\{0.33em}\}=\left\{ \begin{array}{c} @l@\frac{\text{1}}{\text{2}}\varphi_{rep}(\frac{\text{1}}{d\left(x,x_{obs}\right)}-\frac{\text{1}}{d_{\text{0}}}{)}^{\text{2}}\text{\textbackslash{}hspace\{0.33em}\}d(x,x_{obs})\leq d_{\text{0}}\\ 0\text{\textbackslash{}hspace\{0.33em}\;\;\;\;\;\;\;\;\;\;\;\;\;\;\;\;\;\;\;\;\;\;\;\;\;\}d\left(x,x_{obs}\right)>d_{\text{0}} \end{array}\right. \end{array}$$ (8)

$x_{obs}$ is the position of the obstacle, $\varphi_{rep}$ is the repulsive coefficient, $d_{\text{0}}$ is the distance threshold, and $d\left(x,x_{obs}\right)$ is the distance between the node and the obstacle. When the distance $d\left(x,x_{obs}\right)$ between the node and the obstacle is less than or equal to $d_{\text{0}}$, the magnitude of the repulsive force is calculated by the formula; when $d\left(x,x_{obs}\right)$ is greater than $d_{\text{0}}$, the magnitude of the repulsive force is 0. After introducing the artificial potential field method, new nodes are generated along the direction of the attractive force, and their growth vector is:


$$\begin{array}{c} F\left(x\right)\text{\textbackslash{}hspace\{0.33em}\}=\text{\textbackslash{}hspace\{0.33em}\}F_{rand}\left(x\right)+F_{att}\left(x\right)+F_{rep}\left(x\right) \end{array}$$ (9)

At this point, both trees will sample in the direction of F(x) with a fixed step size to obtain new nodes until the two trees are connected, and the search path is completed.

#### 3.1.2. Adaptive step-size environment biasing strategy.

Traditional hybrid RRTs often conflict between global exploration and local obstacle avoidance. In this paper, an adaptive step environmental bias strategy is proposed, which realizes the dynamic synergy of two mechanisms: reducing the step size in the obstacle-dense area to ensure safety, and increasing the step size in the open area to improve efficiency, thus solving the problem that the traditional hybrid algorithm cannot take into account. The adaptive step size environment bias strategy is unique, and when the existing hybrid RRT algorithm encounters complex obstacles, due to the step size limitation, once the search node appears in the obstacle, the search path between the previous node will be directly abandoned, and then the search volume in other directions will be increased, resulting in the search path failure or too long. The adaptive step environment bias strategy avoids this problem, and when the obstacles are complex, the step size will be automatically reduced, which will make it easier to pass through narrow environments and shorten the search path.

The AS-DTRRT algorithm distinguishes itself from traditional RRT and its improved variants through two key innovations. First, it incorporates a target orientation bias mechanism that guides randomly sampled nodes toward target-type convergence, significantly enhancing algorithm convergence speed. Second, it adopts an adaptive step-size environmental bias strategy that dynamically adjusts step lengths in real-time based on local environmental complexity, effectively addressing the core limitations of traditional algorithms: low search efficiency in open environments and high collision risks in complex environments.

The expression of the adaptive step – size environment – biased strategy is as follows:


$$\begin{array}{c} S\left(x\right)=S_{\text{0}}\times \left(1+\delta \times \rho_{step}\right) \end{array}$$ (10)

In this equation, S(x) is the adaptive step size at node x, $S_{\text{0}}$ is the predefined baseline step size, and the step size is adjusted by two core parameters: the environment bias factor δ and the target correction coefficient $\rho_{step}$

To achieve dynamic adjustment of the step size based on environmental complexity, δ is calculated using an exponential decay function, whose expression is as follows:


$$\begin{array}{c} \delta =exp\left(-\frac{N_{obs}}{D_{obs}}\right) \end{array}$$ (11)

$N_{obs}$: The number of obstacle point clouds in the local neighborhood centered at the current node x, which quantifies the obstacle density of the local environment. $D_{obs}$: A preset obstacle density threshold, representing the maximum reasonable number of obstacle point clouds in the neighborhood. δ∈(0,1]: The property of the exponential function ensures that δ monotonically decreases with environmental complexity. When the environment is open, i.e., $N_{obs}\ll D_{obs}$, then δ→1, and the step size approaches its maximum value to maximize search efficiency. When the environment is complex, i.e., $N_{obs}\approx D_{obs}$, then δ→0, and the step size is reduced to near the base step size to ensure obstacle avoidance safety.

To accelerate the convergence of the algorithm toward the target, $\rho_{step}$ is introduced as the target direction bias factor, with a value range of (0,1], and its expression is as follows:


$$\begin{array}{c} \rho_{step}=1-exp\left(-\alpha \cdot \|x-x_{goal}\|\right) \end{array}$$ (12)

In the equation, α is the distance sensitivity coefficient, which regulates the rate at which $\rho_{step}$ changes with the distance to the target. $\|x-x_{goal}\|$ denotes the Euclidean distance between the current node x and the target point $x_{\text{goal}}$.When the current node approaches the target, i.e., $\|x-x_{goal}\|\to 0$, then $\rho_{step}\to 1$, and the step size is enlarged to rapidly converge to the target. When the node is far from the target, $\rho_{step}$ takes a small value, prioritizing the safety of the step size and focusing on environmental exploration.

**Fig 7:**
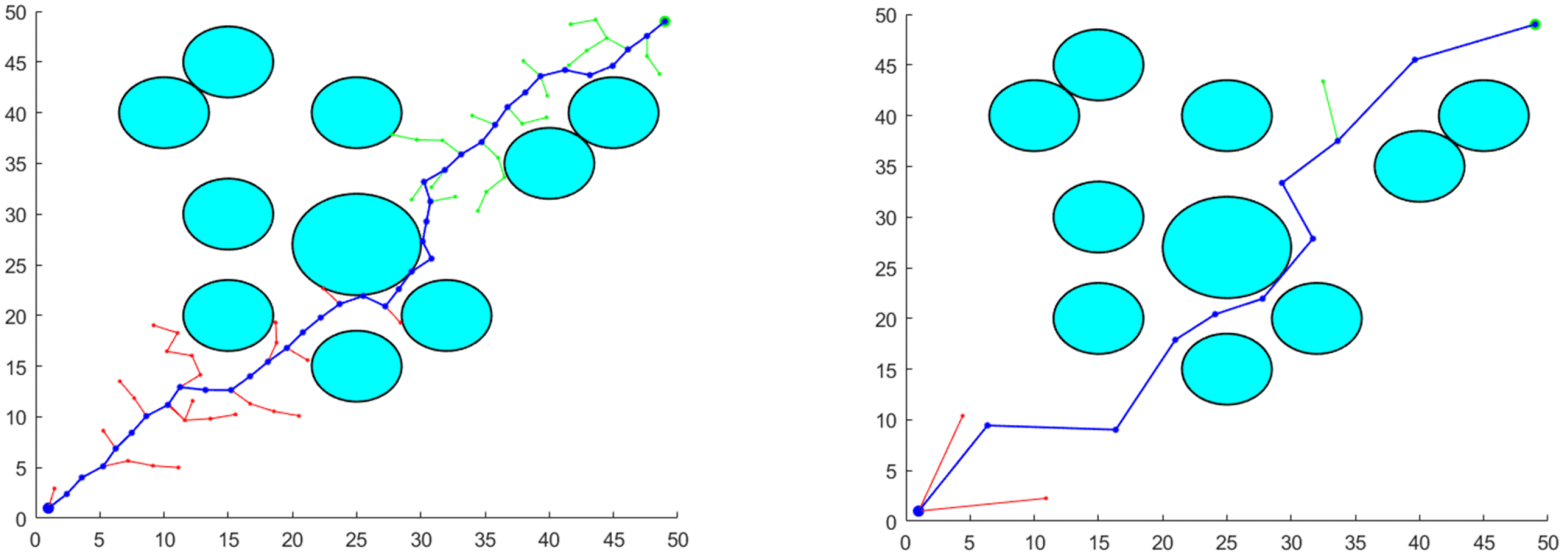
Comparison of different step size strategies. **(a)** Fixed step-size environment biasing strategy; **(b)** Adaptive step-size environment biasing strategy.

Next, a 2D simulation is conducted in MATLAB, and the simulation results are shown in Fig 7. It can be observed from the figure that for the path generated by the adaptive step-size environment bias strategy, the step size is larger when the distance from obstacles is greater and the environment is simple; when the distance from obstacles is smaller and the environment is complex, the step size becomes smaller, making it easier to bypass obstacles. Compared with the fixed-step search strategy, fewer nodes are generated, and the search efficiency is higher. Both algorithms perform searches on the same map with a 50 × 50 mm grid size. The fixed-step environment bias strategy employs 85 search nodes with a path length of 96 mm and a search time of 0.6 seconds, while the adaptive-step environment bias strategy utilizes 14 search nodes, a path length of 92 mm, and a search time of 0.2 seconds. The adaptive-step environment bias strategy outperforms the fixed-step approach in terms of search nodes, path length, and search time efficiency.

#### 3.1.3. Improved Dual-Tree RRT and comparisons with other algorithms.

To verify the feasibility and rapidity of the improved dual-tree RRT algorithm (AS-DTRRT) proposed in this paper, simulations are conducted in MATLAB. Comparisons are made with RRT*, D-RRT, Bi-RRT*, and A-RRT under the same obstacle environment. All algorithms share identical initial nodes, target nodes, and workspace ranges. The initial step size is uniformly set to 10 mm, the maximum number of iterations is 5000, and 50 consecutive searches are performed, with a planning time threshold of 20 s for each search. For the proposed AS-DTRRT algorithm, the distance sensitivity coefficient α is initialized to 0.8, and the target direction bias factor $\rho_{step}$ is initially set to the range (0,1). The core parameters of the comparison algorithms are matched with those of the proposed algorithm to eliminate the influence of initial condition differences on the experimental results. When the number of iterations or the time threshold is exceeded, the search is deemed a failure, and the next search is performed immediately. The experiment terminates when the number of searches reaches 50.

**Fig 8:**
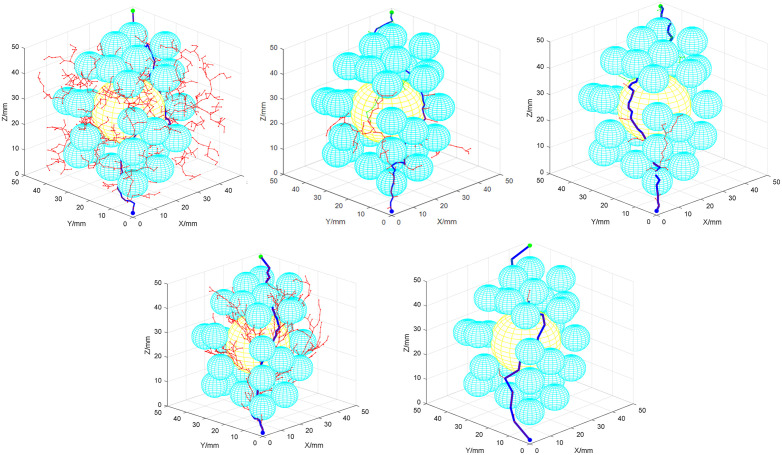
Comparison of different path search algorithms. **(a)** RRT*; **(b)** D-RRT; **(c)** Bi-RRT*; **(d)** A-RRT; **(e)** AS-DTRRT.

**Fig 9:**
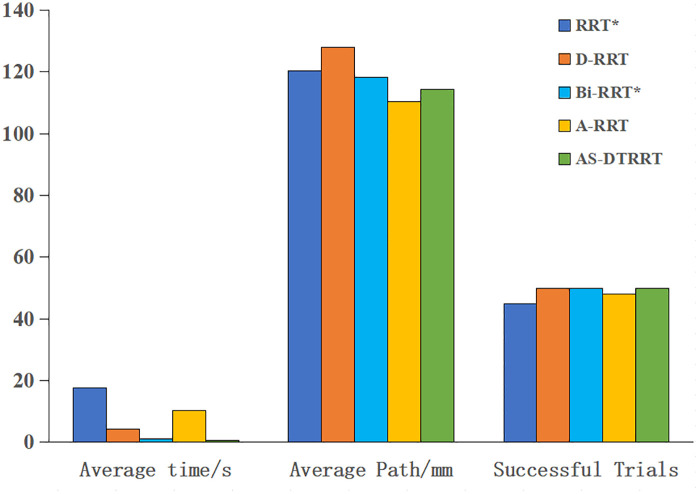
Comparison of different path search algorithms.

The number of successful searches is recorded, and the average search time and path length of successful searches are calculated. The simulation diagram is shown in Fig 8, the simulation results are presented in Table 2, and the variation trends of different algorithms are illustrated in Fig 9.

**Table 2:** Performance indicators of different path search algorithms.

Search algorithms	Average time /s	Average Path /mm	Successful Trials
RRT*	17.661	120.344	45
D-RRT	4.263	127.852	50
Bi-RRT*	1.172	118.326	50
A-RRT	10.364	110.314	48
AS-DTRRT	0.696	114.436	50

As can be seen from the comparison in Fig 8, the AS-DTRRT algorithm generates fewer nodes in its search path, converges faster, reduces the search for blind nodes, and improves search efficiency.

From the data in Table 2, the AS-DTRRT algorithm shows significant advantages in overall performance. Compared with RRT*, the average time of AS-DTRRT is only 0.696 s, which is much lower than RRT*’s 17.661 s; the number of successful searches reaches 50, which is more than RRT*’s 45; and the average path length of 114.436 mm is also shorter. In comparison with D-RRT, AS-DTRRT’s average time of 0.696 s is significantly better than D-RRT’s 4.263 s, with an average path length of 114.436 mm, which is shorter than D-RRT’s 127.852 mm, and both achieve 50 successful searches. Compared to Bi-RRT*, AS-DTRRT has a shorter average time of 0.696 s; although its average path length of 114.436 mm is slightly longer than Bi-RRT*’s 118.326 mm, both achieve the same number of successful searches (50). In contrast to A-RRT, AS-DTRRT’s average time of 0.696 s is much lower than A-RRT’s 10.364 s; it achieves 50 successful searches, which is more than A-RRT’s 48; and its average path length of 114.436 mm is relatively close to A-RRT’s 110.314 mm. Overall, the comprehensive performance of AS-DTRRT is superior to the other four algorithms.

### 3.2. Collision detection

#### 3.2.1. Collision detection for robotic arm.

**Fig 10:**
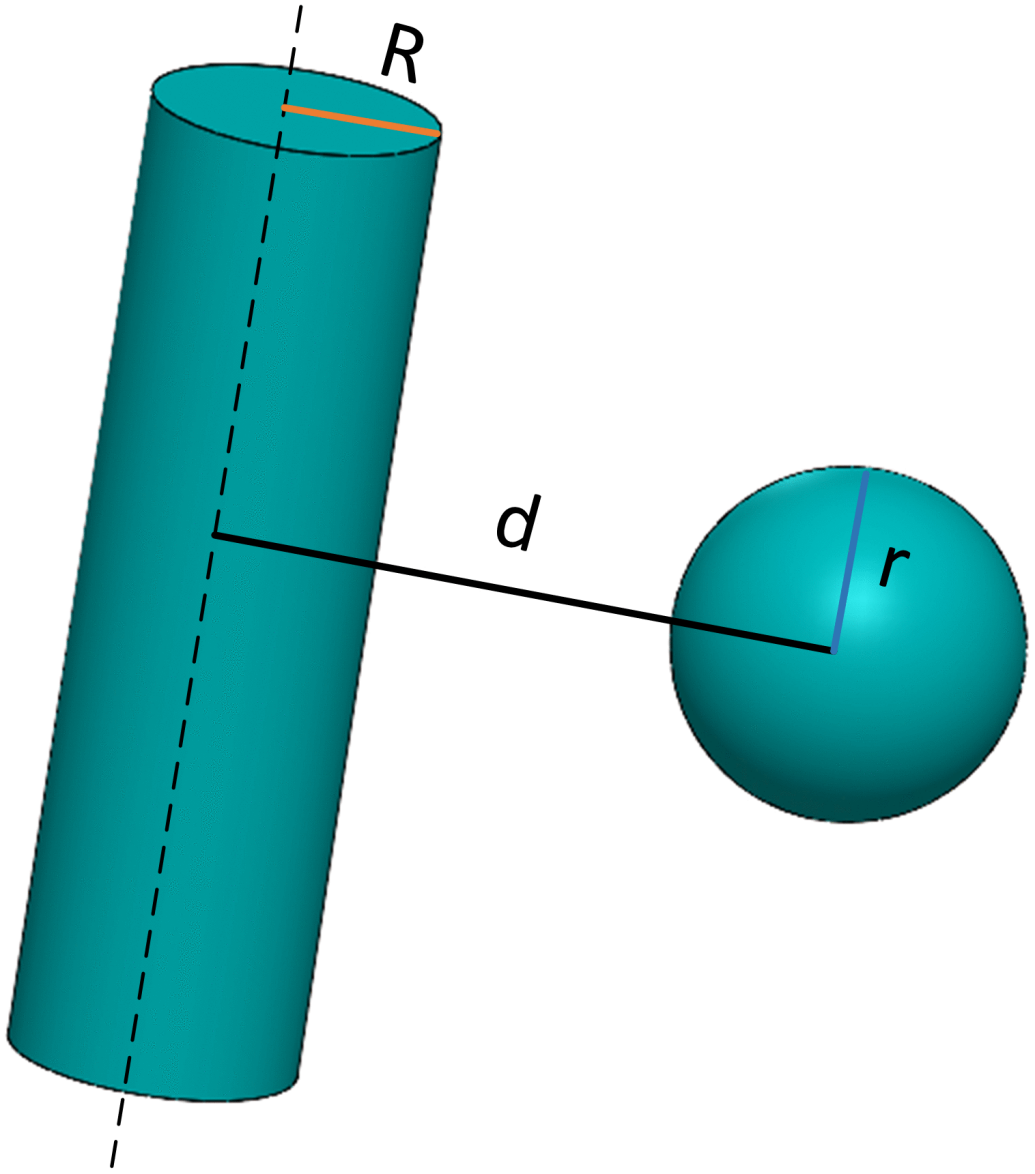
Collision detection model.

In the motion simulation scenario of a robotic arm, to efficiently and accurately determine whether a collision occurs between the robotic arm and obstacles, a specific geometric model is adopted to simplify the problem. Here, the robotic arm is abstracted as a cylindrical bounding box, while obstacles are represented by spherical bounding boxes, as shown in Fig 10. The Euclidean distance method, a commonly used spatial distance measurement approach, can be employed to determine whether a collision occurs between the two. For the cylindrical bounding box, its radius is set as R, i.e., the distance between the cylinder’s axis and its generatrix; the height does not affect the distance judgment here. The radius of the spherical bounding box is r.

Let the center coordinates of the cylinder be $\left(x_{\text{1}},y_{\text{1}},z_{\text{1}}\right)$, and the center coordinates of the sphere be $\left(x_{\text{2}},y_{\text{2}},z_{\text{2}}\right)$. According to the Euclidean distance formula, the distance d between the two centers is:


$$\begin{array}{c} d\text{\textbackslash{}hspace\{0.33em}\}=\text{\textbackslash{}hspace\{0.33em}\}\sqrt{(x_{\text{2}}-x_{\text{1}}{)}^{\text{2}}\text{\textbackslash{}hspace\{0.33em}}+\text{\textbackslash{}hspace\{0.33em}\}(y_{\text{2}}-y_{\text{1}}{)}^{\text{2}}\text{\textbackslash{}hspace\{0.33em}\}+\text{\textbackslash{}hspace\{0.33em}\}(z_{\text{2}}-z_{\text{1}}{)}^{\text{2}}\} \end{array}$$ (13)

The formula for determining whether a collision occurs is as follows:


$$\begin{array}{c} \left\{ \begin{array}{c} @l@d\leq R+r\\ d>R+r \end{array}\right. \end{array}$$ (14)

Whend≤R+r, it can be determined that a collision occurs between the 6-axis robotic arm represented by the cylindrical bounding box and the obstacle represented by the spherical bounding box; conversely, when d>R+r, it indicates that no collision occurs between them. Using the Euclidean distance formula enables rapid and effective real-time monitoring and judgment of collision status between the robotic arm and obstacles, providing an important basis for path planning and safe operation of the robotic arm.

#### 3.2.2. Pipeline collision detection strategies.

When a pipeline installation robot installs pipelines, the robotic arm moves downward from directly above the pipeline to the pipeline position to grasp the pipeline; then it transports the pipeline outside the vehicle for mechanical gripper flipping to keep the pipeline parallel to the wall; finally, it moves to the placement point to complete pipeline placement. Due to the long length of the pipeline, if the pipeline tilts or swings during operation, collisions are likely to occur. Therefore, the pipeline needs to be kept horizontal and parallel to the wall by the mechanical gripper without colliding with environmental obstacles.

**Fig 11:**
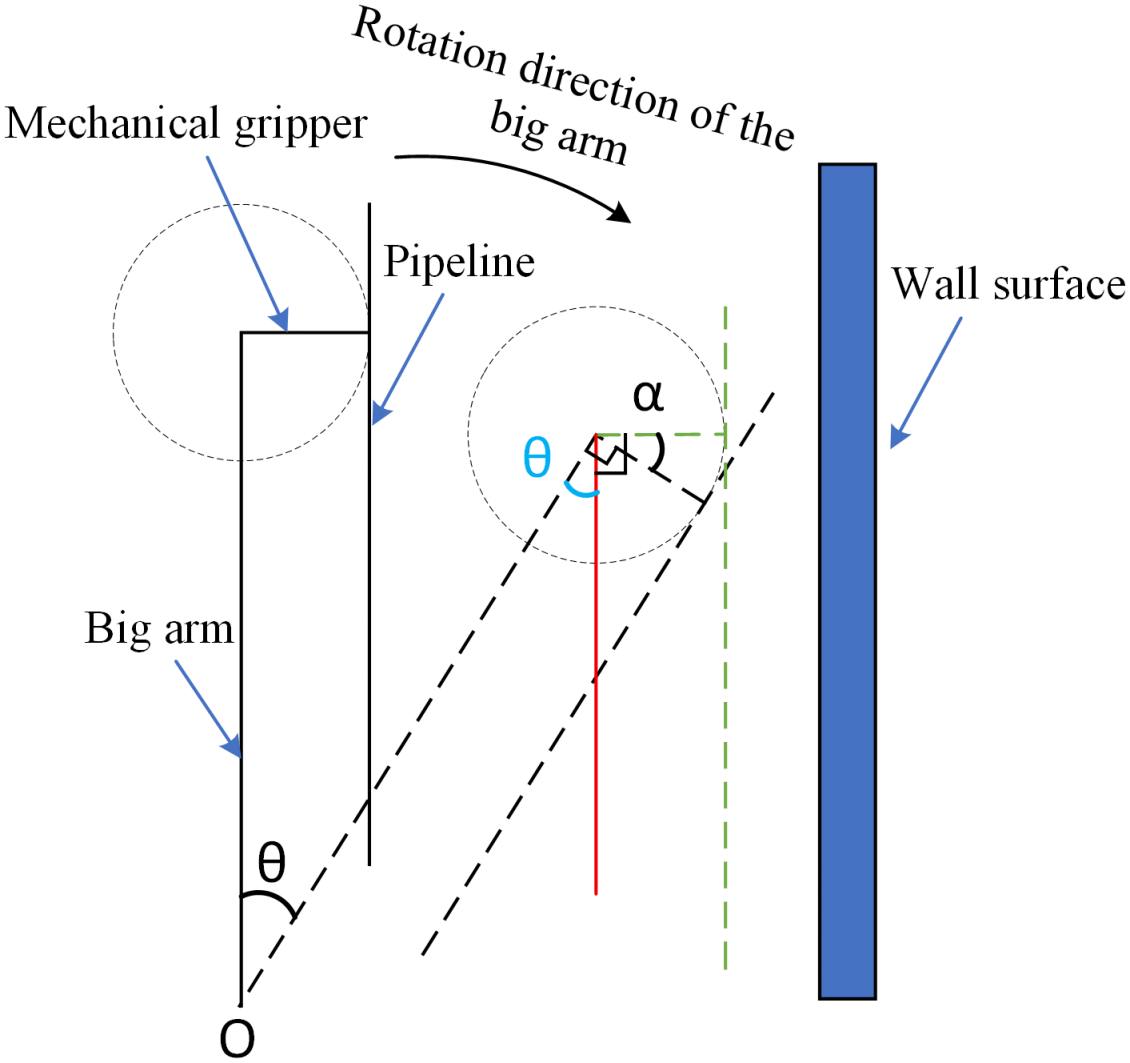
Schematic diagram of robotic arm rotation.

During the movement of the robotic arm grabbing the pipeline, there will be pipeline inclination when the big arm rotates and pitches. When the big arm rotates, the simplified schematic diagram of the robotic arm is shown in Fig 11. The pipeline is kept parallel to the wall. Suppose the big arm rotates around point O by an angle θ to reach the dashed position; at this time, the pipeline is not parallel to the wall, which is not conducive to pipeline installation. In order to keep the pipeline balanced with the wall during movement, the mechanical gripper should rotate by an angle α to reach the blue dashed position. Both motions are performed simultaneously and take the same amount of time.

Therefore, the rotational angular velocity$\omega_{\text{1}}$ of the big arm and the rotational angular velocity$\omega_{\text{4}}$ of the wrist 2 should satisfy the following formula:


$$\begin{array}{c} \omega_{\text{1}}\text{\textbackslash{}hspace\{0.33em}\}=\text{\textbackslash{}hspace\{0.33em}\}\frac{\Delta \theta }{\Delta \alpha }\omega_{\text{4}} \end{array}$$ (15)

By analyzing the above figure and drawing the red auxiliary line, it can be concluded that θ\hspace{0.33em}=\hspace{0.33em}α, and thus:


$$\begin{array}{c} \omega_{\text{1}}\text{\textbackslash{}hspace\{0.33em}\}=\text{\textbackslash{}hspace\{0.33em}\}\omega_{\text{4}} \end{array}$$ (16)

**Fig 12:**
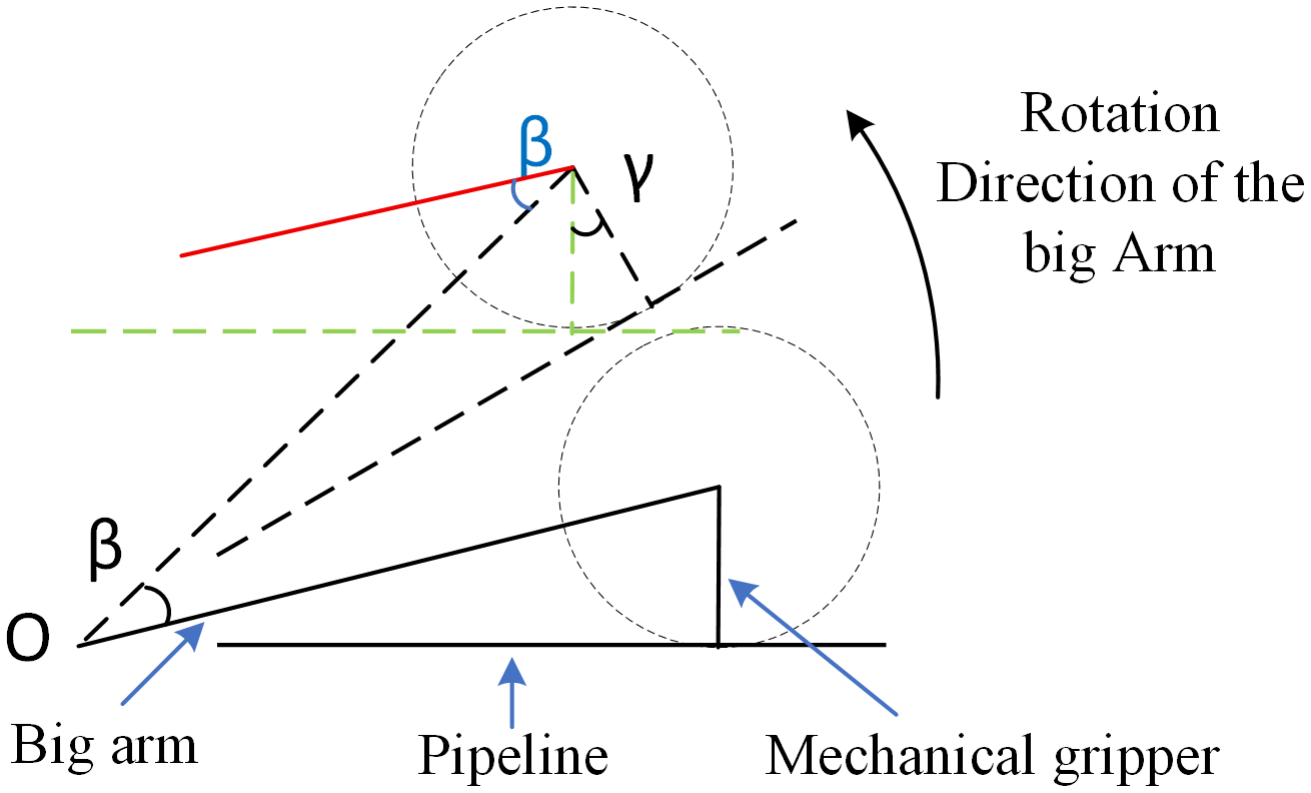
Schematic diagram of robotic arm pitching.


When the big arm pitches, the schematic diagram of the robotic arm pitching is shown in Fig 12. The pipeline is in a horizontal state. Suppose the big arm rotates around point O by an angle β to reach the dashed position; at this time, the pipeline tilts, which is not conducive to pipeline installation. To keep the pipeline horizontal during movement, the mechanical gripper should rotate by an angle γ to reach the green dashed position. Both motions are performed simultaneously and take the same amount of time. Therefore, the pitching angular velocity $\omega_{\text{2}}$ of the big arm and the rotational angular velocity $\omega_{\text{4}}$ of the wrist 2 should satisfy the following formula:

$$\begin{array}{c} \omega_{\text{2}}\text{\textbackslash{}hspace\{0.33em}\}=\text{\textbackslash{}hspace\{0.33em}\}\frac{\Delta \beta }{\Delta \gamma }\omega_{\text{4}} \end{array}$$ (17)

Analyzing the above figure and drawing the red auxiliary line, it can be concluded that β=γ, and thus:


$$\begin{array}{c} \omega_{\text{2}}\text{\textbackslash{}hspace\{0.33em}\}=\text{\textbackslash{}hspace\{0.33em}\}\omega_{\text{4}} \end{array}$$ (18)

From the above analysis, it can be concluded that when the big arm rotates, the rotational angular velocity of the big arm and that of wrist 2 should be equal to keep the pipeline parallel to the wall. When the big arm pitches, the pitching angular velocity of the big arm and the rotational angular velocity of wrist 2 should be equal to keep the pipeline in a horizontal state. When both situations occur simultaneously, the working condition requirements can be met as long as the three angular velocities are equal.

**Fig 13:**
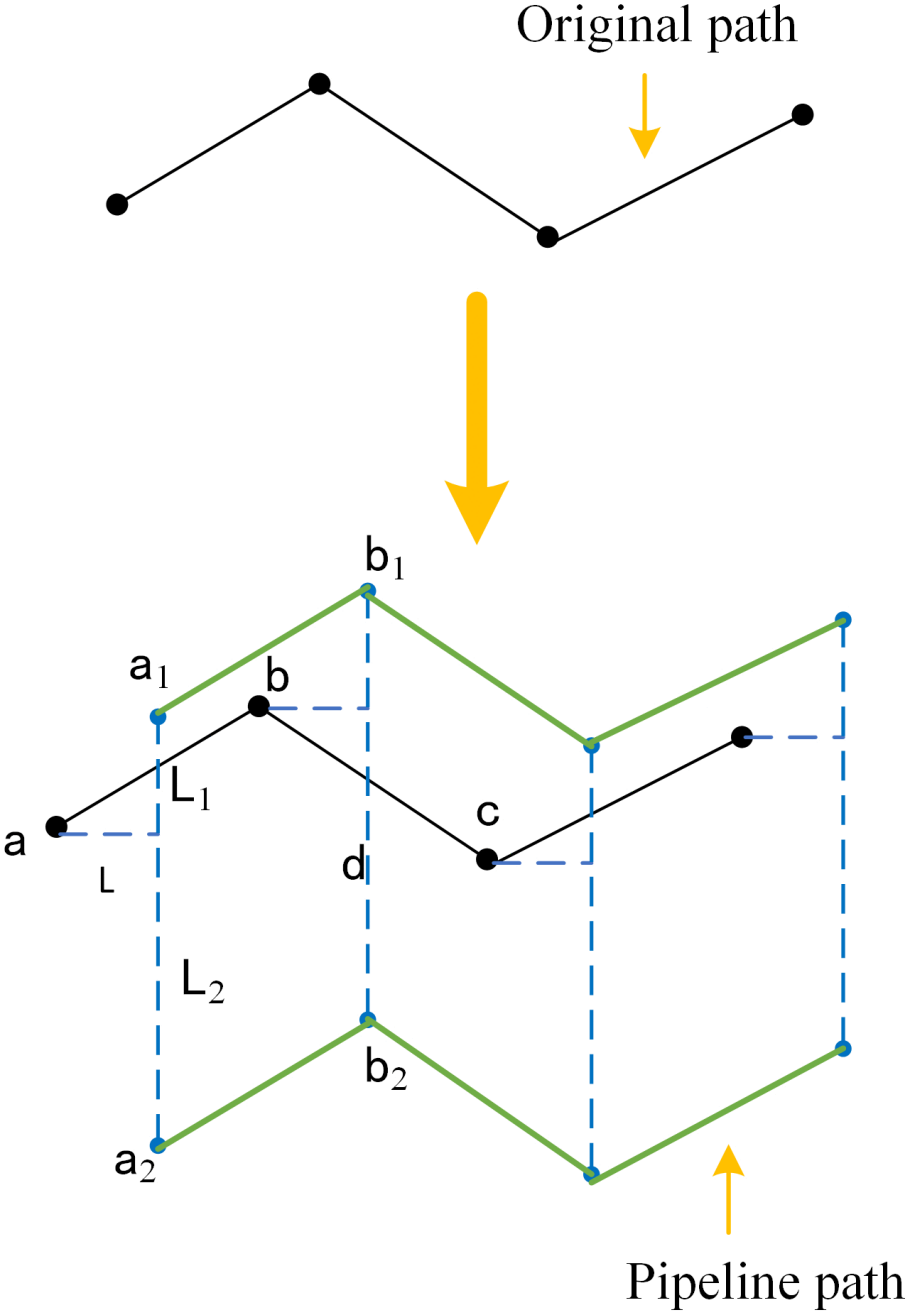
Original path and actual pipeline path.

Through working condition analysis, by coordinating the angular velocities of different joints, the pipeline can be maintained in a horizontal state and parallel to the wall. The original path searched by the improved dual-tree RRT algorithm can only ensure that the path and the robotic arm do not collide with environmental obstacles. Due to the length of the pipeline, there is a difference between the actual path of the pipeline and the original path, and the specific situation is shown in Fig 13.

In Fig 13, the black line represents the original path, the blue dashed line represents the actual position of the pipeline, and the green line represents the actual path of the pipeline; a, b, c, d represent path points, a_1_ and a_2_ represent the path points at both ends of the pipeline under point a, and the same applies to b_1_ and b_2_. Suppose the coordinates of point a are (x_1_ + L, y_1_ + L_1_, z_1_), then a_1_ and a_2_ are (x_1_ + L, y_1_ + L_1_, z_1_) and (x_1_ + L, y_1_-L_2_, z_1_) respectively. Collision detection is performed on a_1_ and a_2_ using the Euclidean geometric distance: first, workspace detection is carried out to check whether both ends of the pipeline exceed the workspace; then, collision detection between the pipeline and environmental obstacles is performed. If both points pass the collision detection, the original path point a is retained. By analogy, through backtracking the path, pipeline collision detection is performed on the entire path, path points that meet the conditions are retained, and those that do not meet the conditions are eliminated. The path is searched again, and pipeline collision detection is performed until the process is completed.

#### 3.2.3. Limitations of collision models and dynamic risk analysis.

In this paper, the collision detection model is simplified using the bounding box method to reduce computational complexity and ensure real-time performance. Although this method has strong generality, it still has certain limitations in the scenario of long pipeline installation. The coal mine tunnel environment is complex and changeable, and the above simplification may lead to deviations in practical applications. In particular, during the movement of the manipulator carrying a long pipeline, the pipeline is prone to swinging, twisting and other phenomena, resulting in potential collision risks.

At present, the pipeline obstacle avoidance strategy proposed in this paper is mainly analyzed from the perspective of position constraints and motion coordination, where the posture of the long pipeline is guaranteed by adjusting the angular velocities of each joint of the manipulator. Although the scheme is feasible, it still has deficiencies, and a complete spatial collision model integrating the pipeline, robot and tunnel has not been established. To realize quantitative analysis of dynamic collision risks, future research will introduce finite element analysis (FEA) or dynamic modeling of flexible pipelines, and incorporate dynamic characteristics such as pipeline vibration amplitude, torsion angle and deformation range into the existing model. In this way, collision risks can be more comprehensively evaluated under complex working conditions, further improving the safety and reliability of the robot’s obstacle avoidance strategy.

### 3.3. Trajectory planning

#### 3.3.1. Quintic polynomial interpolation.

The fifth – order polynomial interpolation method is used to generate smooth and continuous motion trajectories in robotic arm trajectory planning. To ensure the smoothness of the operation trajectory of the pipeline installation robot and reduce impacts, etc., the fifth – order polynomial interpolation is selected for trajectory planning. Assuming that the starting point $p_{\text{0}}$, starting velocity $v_{\text{0}}$, starting acceleration $a_{\text{0}}$, end point $p_{f}$, end velocity $v_{f}$, and motion time t are given, the expression of the fifth – order polynomial interpolation is as follows:


$$\begin{array}{c} p\left(t\right)\text{\textbackslash{}hspace\{0.33em}\}=\text{\textbackslash{}hspace\{0.33em}\}b_{\text{0}}\text{\textbackslash{}hspace\{0.33em}\}+\text{\textbackslash{}hspace\{0.33em}\}b_{\text{1}}t+\text{\textbackslash{}hspace\{0.33em}\}b_{\text{2}}t^{\text{2}}+\text{\textbackslash{}hspace\{0.33em}\}b_{\text{3}}t^{\text{3}}+\text{\textbackslash{}hspace\{0.33em}\}b_{\text{4}}t^{\text{4}}+\text{\textbackslash{}hspace\{0.33em}\}b_{\text{5}}t^{\text{5}} \end{array}$$ (19)

where t is the time variable, and $b_{\text{0}}$, $b_{\text{1}}$, $b_{\text{2}}$, $b_{\text{3}}$, $b_{\text{4}}$, and $b_{\text{5}}$ are the polynomial coefficients to be determined.

By taking the first – order derivative and the second – order derivative of p(t), the formulas for velocity and acceleration can be obtained:


$$\begin{array}{c} \left\{ \begin{array}{c} @l@p^{\prime }\left(t\right)\text{\textbackslash{}hspace\{0.33em}\}=\text{\textbackslash{}hspace\{0.33em}\}b_{\text{1}}\text{\textbackslash{}hspace\{0.33em}\}+\text{\textbackslash{}hspace\{0.33em}\}2b_{\text{2}}t+\text{\textbackslash{}hspace\{0.33em}\}3b_{\text{3}}t^{\text{2}}+\text{\textbackslash{}hspace\{0.33em}\}4b_{\text{4}}t^{\text{3}}+\text{\textbackslash{}hspace\{0.33em}\}5b_{\text{5}}t^{\text{2}}\\ p^{\prime \prime }\left(t\right)\;=\text{\textbackslash{}hspace\{0.33em}\}2b_{\text{2}}+\text{\textbackslash{}hspace\{0.33em}\}6b_{\text{3}}t+\text{\textbackslash{}hspace\{0.33em}\}12b_{\text{4}}t^{\text{2}}+\text{\textbackslash{}hspace\{0.33em}\}20b_{\text{5}}t^{\text{3}}\\ v\left(t\right)\;=\text{\textbackslash{}hspace\{0.33em}\}p^{\prime }\left(t\right)\\ a\left(t\right)\text{\textbackslash{}hspace\{0.33em}\}\;=\text{\textbackslash{}hspace\{0.33em}\}p^{\prime \prime }\left(t\right) \end{array}\right. \end{array}$$ (20)

To prevent planned trajectories from exceeding the physical capabilities of hydraulic drive systems, this study introduces strict constraints on joint velocity and acceleration during the fifth-order polynomial trajectory smoothing process. Rotational speeds of individual joints are limited to 20 degrees per second (°/s), while acceleration is capped at 20 m/s², ensuring the interpolated trajectories remain within the dynamic response range of hydraulic systems. Angular encoders are installed on rotating joints and displacement sensors on moving joints, enabling real-time recording of joint position and angle parameters to facilitate stable trajectory tracking by the robot.

#### 3.3.2. Pipeline constraints.

After the robotic arm grabs the pipeline, the pipeline will affect trajectory planning. For example, the length and weight of the pipeline will restrict the running trajectory of the robotic arm and the running speed of each joint. It is necessary to consider the pipeline and the end of the robotic arm as a whole. Therefore, it is necessary to establish pipeline constraints, mainly including pipeline geometric obstacle – avoidance constraints and load constraints.

(1) Pipeline geometric obstacle-avoidance constraints:

$$\begin{array}{c} \left\{ \begin{array}{c} @l@|p(t)-p_{\text{pipe}}|\geq \frac{d}{\text{2}}+\varepsilon \text{\textbackslash{}hspace\{1em}\}(t<t_{p})\\ p_{\text{pipe, end}}\text{\textbackslash{}hspace\{0.33em}\}\left(t\right)\notin O\text{\textbackslash{}hspace\{1em}\}(t\geq t_{p}) \end{array}\right. \end{array}$$ (21)

In Formula (21), $p_{\text{pipe}}$ represents the position of the pipeline axis, d represents the diameter of the pipeline, ε represents the safety clearance between the robotic arm and the pipeline, t represents the running time of the robotic arm, $t_{p}$ represents the time when the robotic arm grabs the pipeline, $p_{\text{pipe, end}}$ represents the positions of both ends of the pipeline, and O represents the obstacle region. When $t<t_{p}$, the robotic arm has not grabbed the pipeline, and it needs to be greater than the sum of the pipeline radius and the safety clearance to prevent collision; when $t\geq t_{p}$, the robotic arm grabs the pipeline, and it is ensured that both ends of the pipeline are not in the obstacle region.

Load constraint:


$$\begin{array}{c} \left\{ \begin{array}{c} @l@\left|p^{\prime }\left(t\right)\right|\leq v_{\text{max}}\cdot \left(1-\frac{m}{m_{\text{max}}}\right)\\ \left|p^{\prime \prime }\left(t\right)\right|\leq a_{\text{max}}\cdot \left(1-\frac{m}{m_{\text{max}}}\right) \end{array}\right. \end{array}$$ (22)

In Formula (22), $v_{\text{max}}$ represents the maximum operating speed of the robotic arm under no – load condition, $a_{\text{max}}$ represents the maximum acceleration of the robotic arm under no – load condition, m represents the mass of the pipeline, and $m_{\text{max}}$ represents the maximum mass that the robotic arm can bear. When m =0, it means the robotic arm is under no – load condition. The greater the load of the robotic arm, the smaller the maximum operating speed and acceleration, so as to avoid overload damage of the robotic arm.

By substituting the above constraint conditions, the coefficients $b_{\text{0}}$, $b_{\text{1}}$, $b_{\text{2}}$, $b_{\text{3}}$, $b_{\text{4}}$, and $b_{\text{5}}$ can be solved. The fifth – order interpolation polynomial has continuous second – order derivatives, that is, the acceleration also changes continuously, which makes the motion trajectory smoother and reduces vibration and impact.

#### 3.3.3. Trajectory planning model.

**Fig 14:**
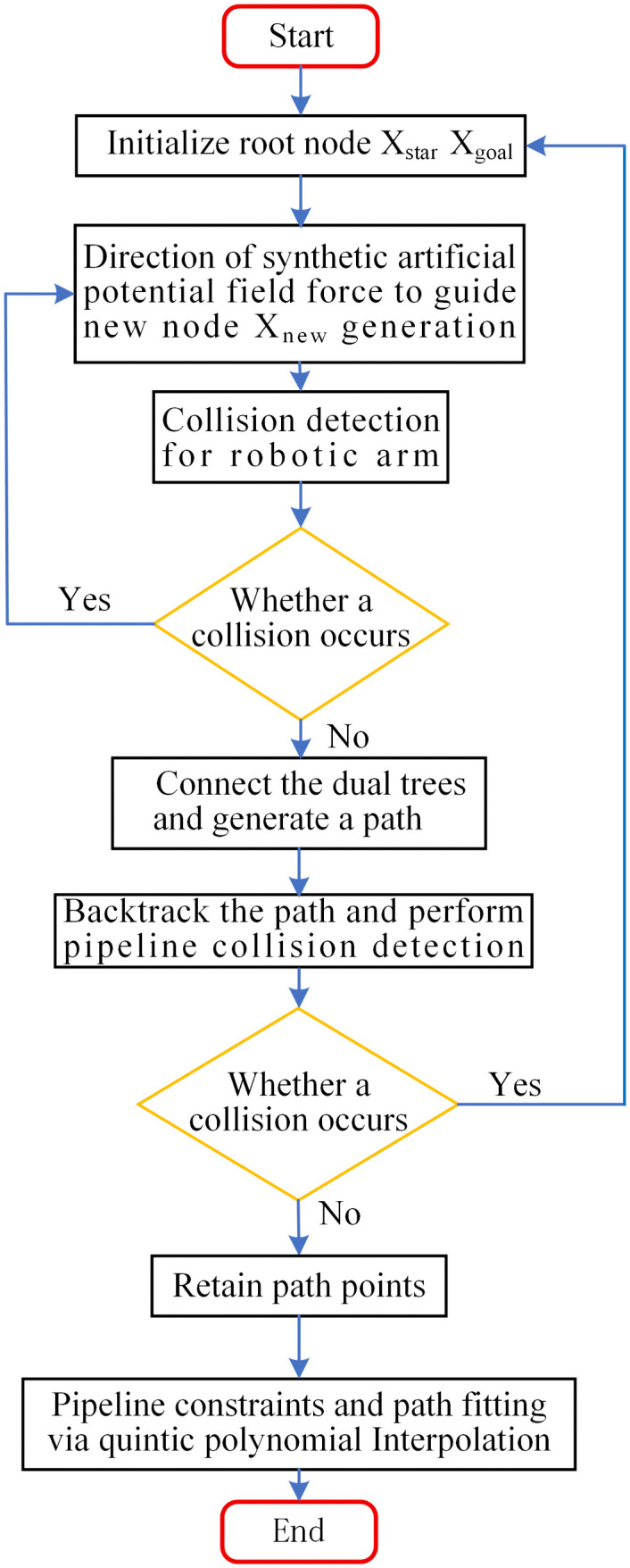
Trajectory planning model.

Fig 14 illustrates the trajectory planning model. First, an initial collision-free path between the robotic arm and the environment is planned using the improved dual-tree RRT algorithm. On this basis, pipeline collision detection is performed to obtain a collision-free path that meets the working conditions of the pipeline installation robot. Finally, polynomial interpolation is used for trajectory planning.

## 4. Experiments

### 4.1. Simulation experiment test analysis

**Fig 15:**
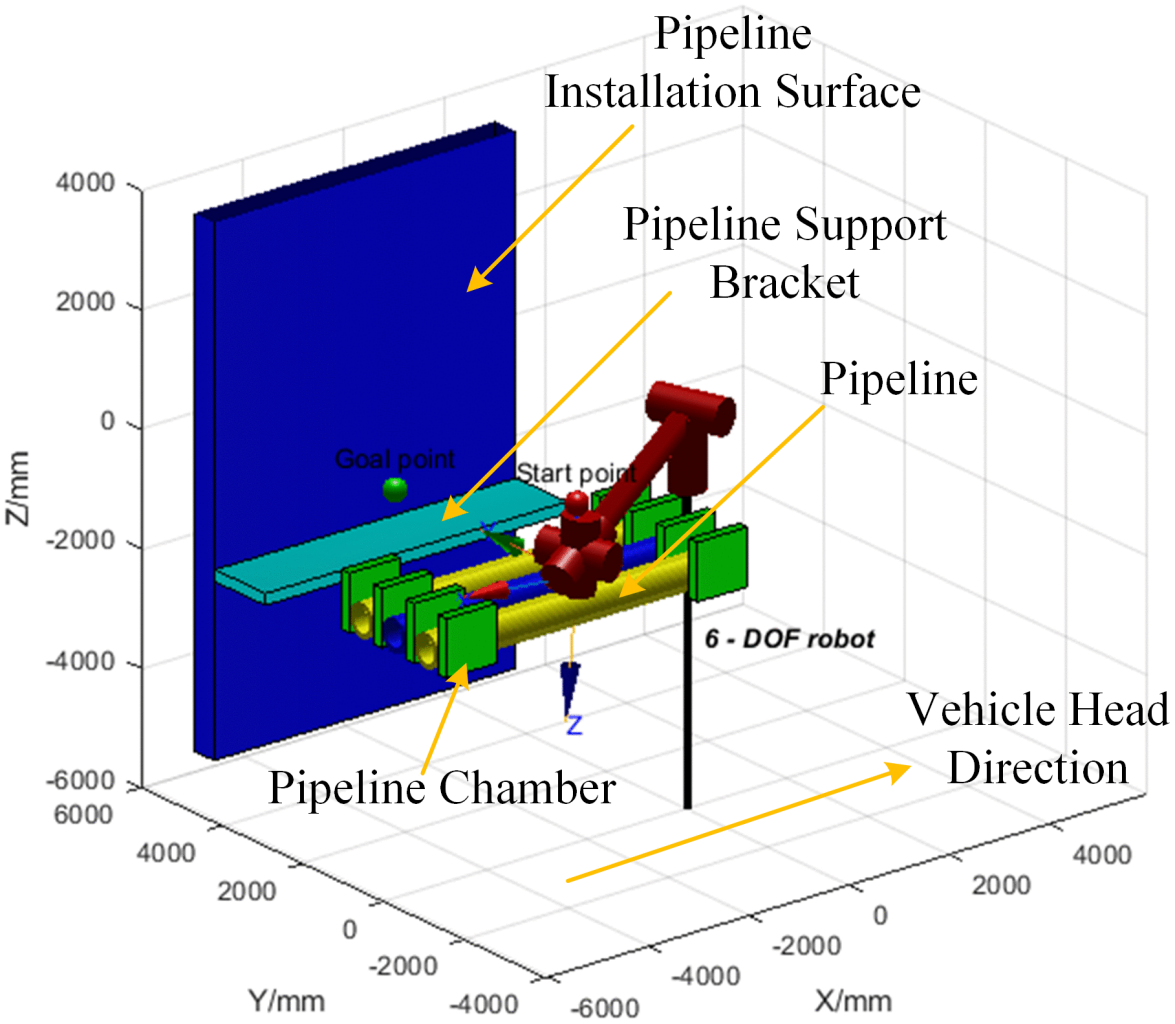
Simulation of robotic arm and environment.

**Fig 16:**
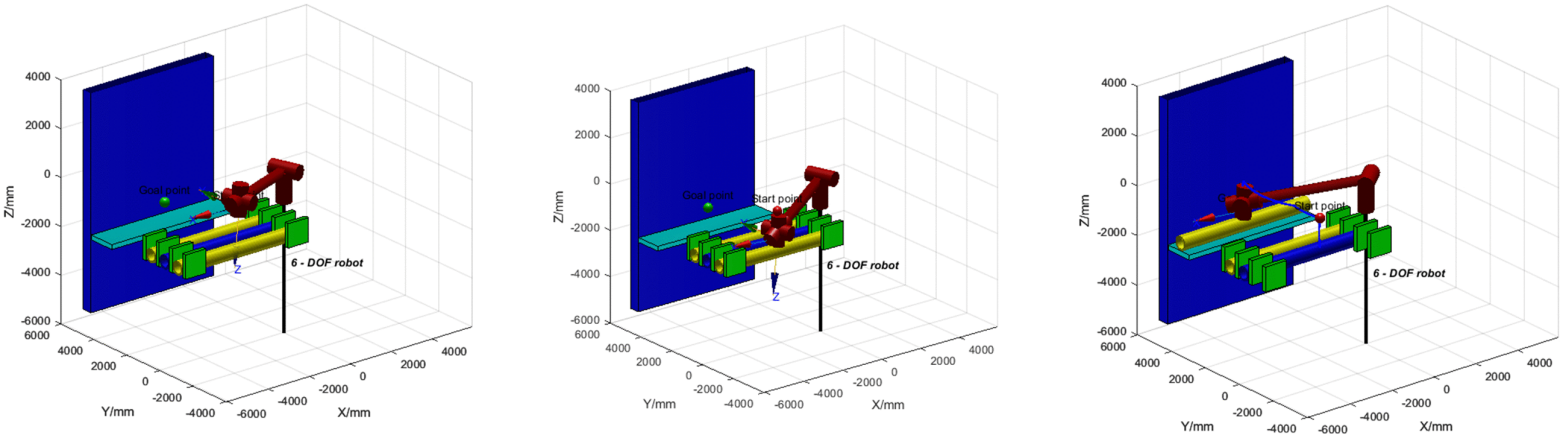
Simulation of robotic arm movement process. **(a)** The robotic arm is at the starting position; **(b)** The robotic arm is at the grasping position; **(c)** The robotic arm is at the target position.

**Fig 17:**
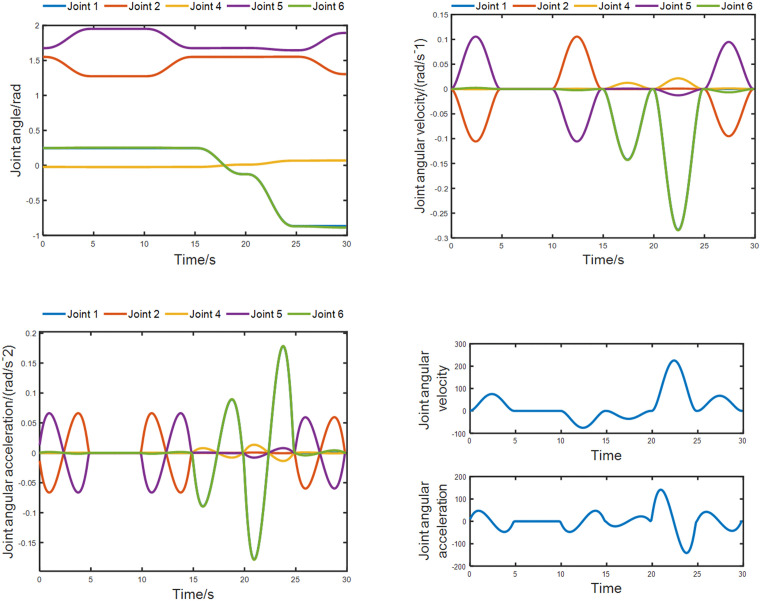
Parameter curves of joint in trajectory planning. **(a)** Rotational joint angle vs. time curve; **(b)** Rotational joint angular velocity vs. time curve; **(c)** Rotational joint angular acceleration vs. time curve; **(d)** Parameters of prismatic joint 3.

In MATLAB, the obstacle environment and the robotic arm are established, and the force of the mechanical gripper clamping the pipeline is simulated. Environmental constraints and pipeline constraints are added to ensure no collision occurs. The spatial configuration measures 5800 mm in length, 6000 mm in width, and 4800 mm in height. The pipeline dimensions include a length of 4000 mm, a diameter of 150 mm, a wall thickness of 20 mm, and a weight of 200 kg. To facilitate observation, the simulation simplified the robot’s main body model for pipeline installation, retaining key components such as the robotic arm, pipelines, and roadway surfaces. These elements were excluded from the robotic arm’s operational range to ensure no interference with its actual performance, as shown in Fig 15. Then, the AS-DTRRT search algorithm is used to perform path search first, and finally, trajectory planning is carried out through fifth-order polynomial interpolation. The simulation of the robotic arm movement process is shown in Fig 16. The robotic arm first moves downward from the starting point to the grasping point to grasp the pipeline, then moves back to the position of the starting point to ensure a safe movement height, continues to move to the flipping point to flip the mechanical gripper to ensure it is parallel to the installation surface, and finally moves to the placement point to complete the trajectory. The simulated trajectory is shown in Fig 17.

During the movement, the variation curves of the motion parameters of each joint are shown in Fig 17. Among them, Fig (a), Fig (b), and Fig (c) are the variation curves of the angles, angular velocities, and angular accelerations of the five rotational joints with time, respectively; Fig (d) is the variation curve of the velocity and acceleration of the translational joint 3 with time.

It can be analyzed that from the curve graphs of joint angles versus time, the variation curves of each joint angle are very smooth, and there are no cusps at the transition points, which meets the requirements of trajectory planning. In the curve graphs of angular accelerations of rotational joints versus time, the velocity curves of Joint 2, Joint 5, and Joint 6 show oscillation phenomena, indicating that the velocity changes sharply. This is because the pipeline installation robot is set to a linkage mode, and not all six axes can move simultaneously, which causes the velocity of the joint axes to start and stop frequently. However, from the perspective of each segment of change, the velocity curve changes smoothly, and there are no cusps at the turning points, which meets the requirements. Similarly, the angular accelerations of the rotational joints are also like this. The variation curves of the velocity and acceleration of Joint 3 (translational joint) are smooth, which meets the requirements. Thus, it can be proved that the trajectory planning strategy can meet the motion requirements of the pipeline installation robot.

### 4.2. Experimental test analysis of pipeline installation robots

**Fig 18:**
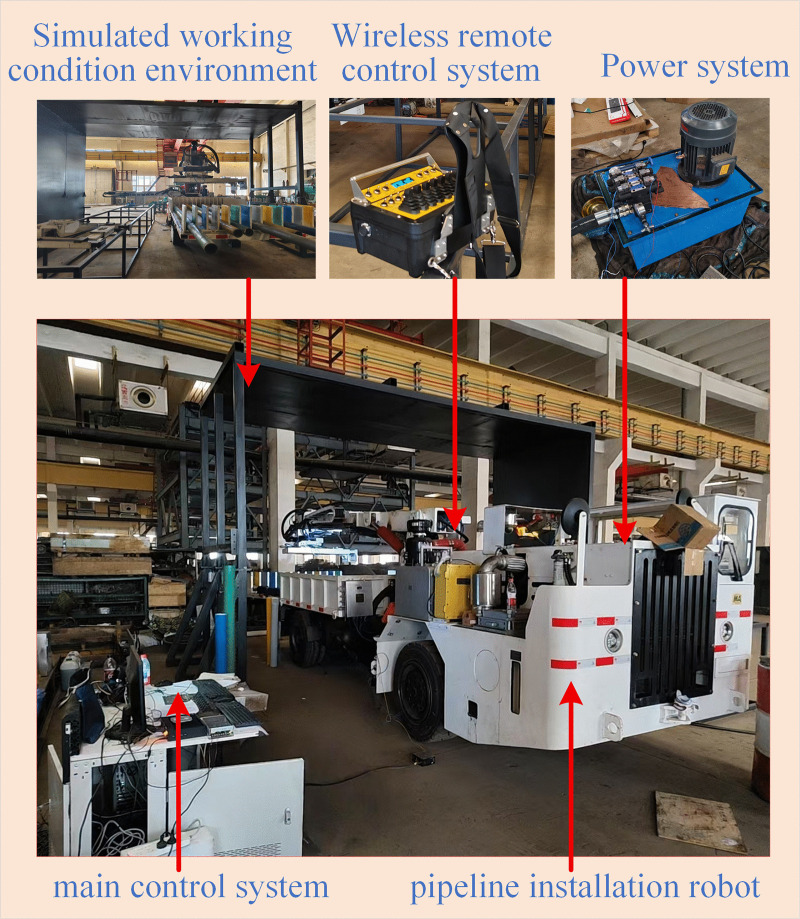
Experimental platform of pipeline installation robot.

To verify the engineering practicability of the AS-DTRRT algorithm on the on-board embedded platform of the pipeline installation robot, a working environment highly restored to the underground coal mine pipeline installation scenario is built on a 1:1 scale, namely the pipeline installation robot experimental platform, as shown in Fig 18. Deployment tests are carried out based on the on-board embedded main control system shown in Fig 18. As the core computing unit of the robot, the main control system directly runs the proposed algorithm to drive the robot to complete pipeline installation tasks.

(1) Hardware Composition of the Experimental Platform

The experimental platform consists of five modules: pipeline installation robot, simulated working condition environment, main control system, wireless remote control system, and power system. The configuration of each part is as follows:

Pipeline installation robot: The main body has dimensions of 5200 mm in length and 3800 mm in width, driven by a hydraulic chassis. It is equipped with a 6-degree-of-freedom (6-DOF) heavy-duty manipulator for grasping, posture adjustment and installation of long pipelines. A 3D LiDAR is integrated on the vehicle for environmental perception and obstacle detection.

Simulated working condition environment: Built in a 1:1 scale of the underground coal mine roadway. The roadway has a width of 6000 mm and a height of 4800 mm, and its length is much larger than the vehicle length, which has no impact on the experiment and is thus not listed. The pipeline has a length of 4000 mm, a diameter of 150 mm, a wall thickness of 20 mm, and a weight of 200 kg, which fully reproduces the real working scenario of underground pipeline installation.

Main control system: Composed of a mine-used flameproof and intrinsically safe vehicle-mounted controller, an industrial computer and a display screen. The industrial computer model is IPC610, responsible for running the trajectory planning algorithm; the display screen model is TF-G270.

Power system: An independent hydraulic pump station is adopted to provide hydraulic power for the robot chassis travel and manipulator joint movement. The system operating pressure is 16 MPa with a flow rate of 30 L/min.

Wireless remote control system: A mine-used intrinsically safe remote controller is used, with a communication distance of more than 30 m. It supports one-button grasping, one-button placement, emergency stop and other operations, realizing remote control and operation triggering of the robot.

(2) Key Parameter Settings

The maximum joint angular velocity of the manipulator’s rotary joints is 20°/s, and the maximum joint angular acceleration is 20°/s²; the maximum moving speed of the prismatic joints is 250 mm/s, and the maximum acceleration is 250 mm/s².

The system control cycle is 100 ms, and the data acquisition frequency is 10 Hz.

In the figure, the dimensions of the pipeline installation robot are 5200 mm in length and 3800 mm in width. The hydraulic power unit provides power for the entire hydraulic system. Through the one-click grabbing and one-click placing functions of the wireless remote controller, the robot can automatically complete the pipeline installation according to the planned trajectory.

**Fig 19:**
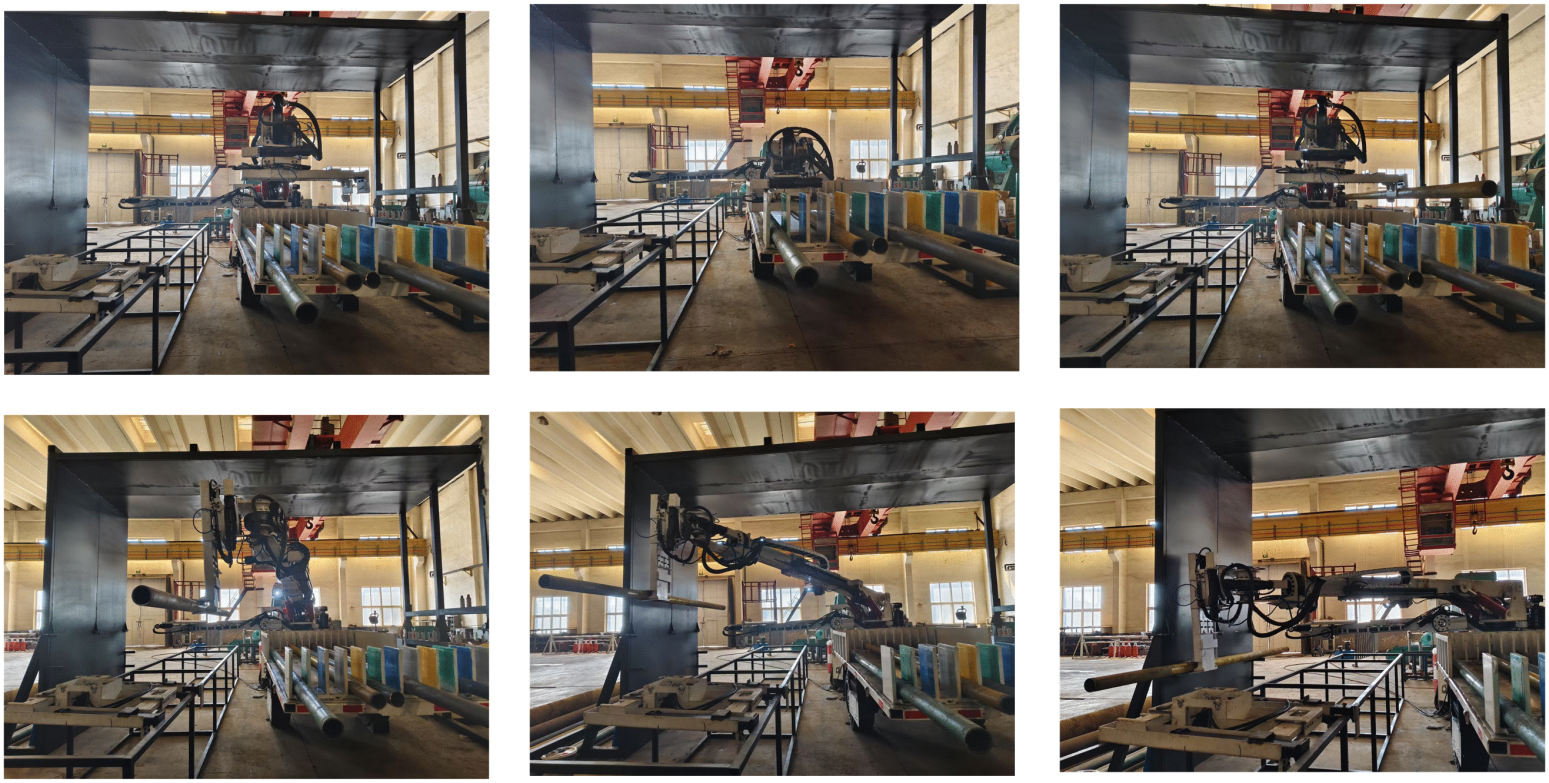
Actual movement process of pipeline installation robot. (a) a_1_ point; (b) c_1_ point和c_2_point; (c) a_2_ point; (d) f point; (e) d point; (f) e point.

Fig 19 shows the experimental test of the pipeline installation robot. The entire trajectory includes points a_1_, c_1_, c_2_, a_2_, f, d, and e. Among these, a_1_ and a_2_, as well as c_1_ and c_2_, are the same points but are distinguished by different names due to the different trajectory movement directions. Point a_1_ is the starting point, and point e is the target point. The robotic arm first moves downward from a_1_ to c_1_ to grab the pipeline; then moves upward from c_2_ to a_2_; next moves from a_2_ to f, where the mechanical gripper is flipped to remain parallel to the installation surface; then moves from f to d; and finally moves from d to e (the placement point), marking the end of the trajectory. During the movement, the operation was stable without collisions with obstacles, showing no discrepancy from the simulation results. This meets the working requirements and verifies the rationality of the trajectory planning.

**Fig 20:**
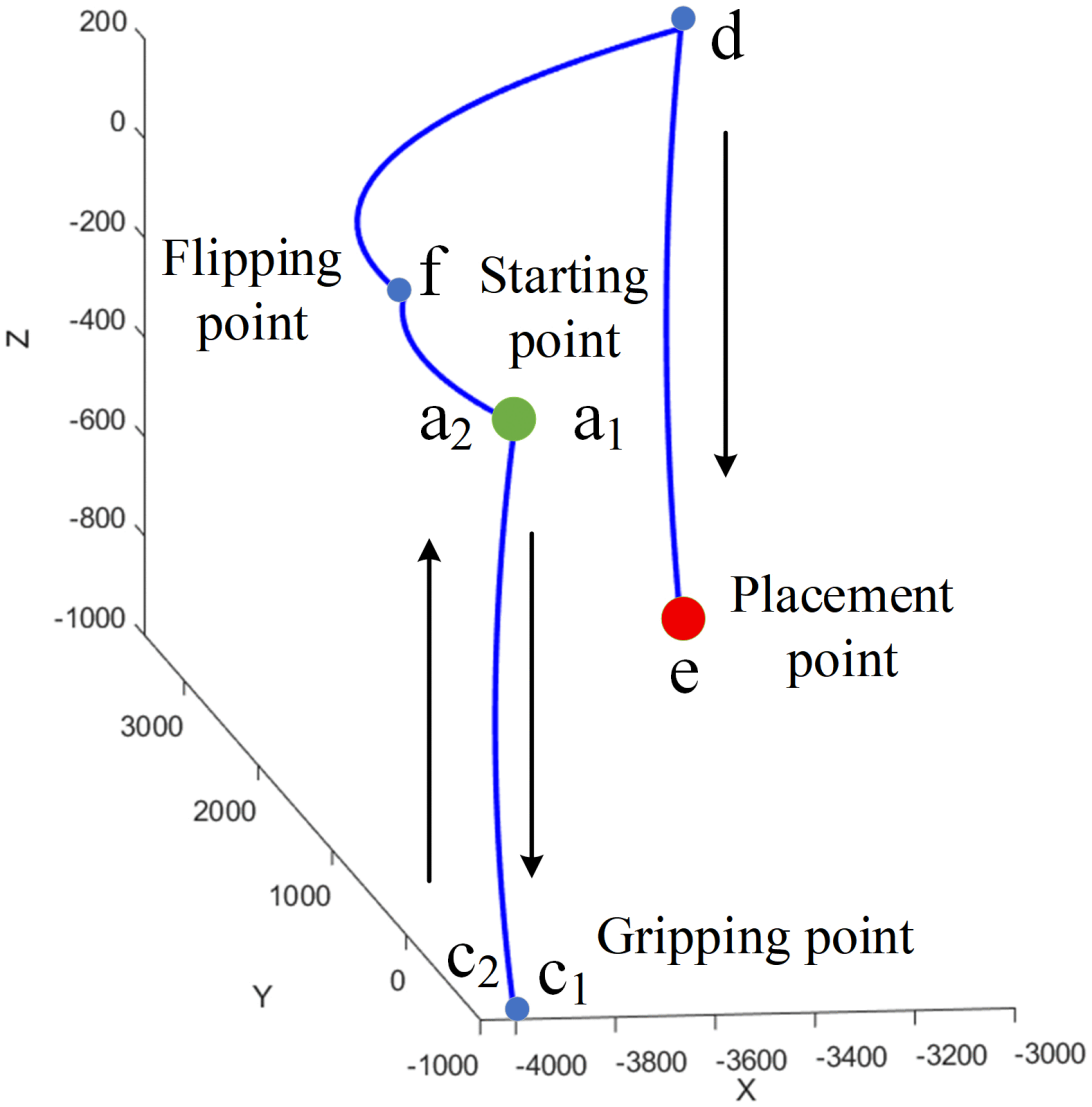
Movement trajectory of robotic arm.

**Fig 21:**
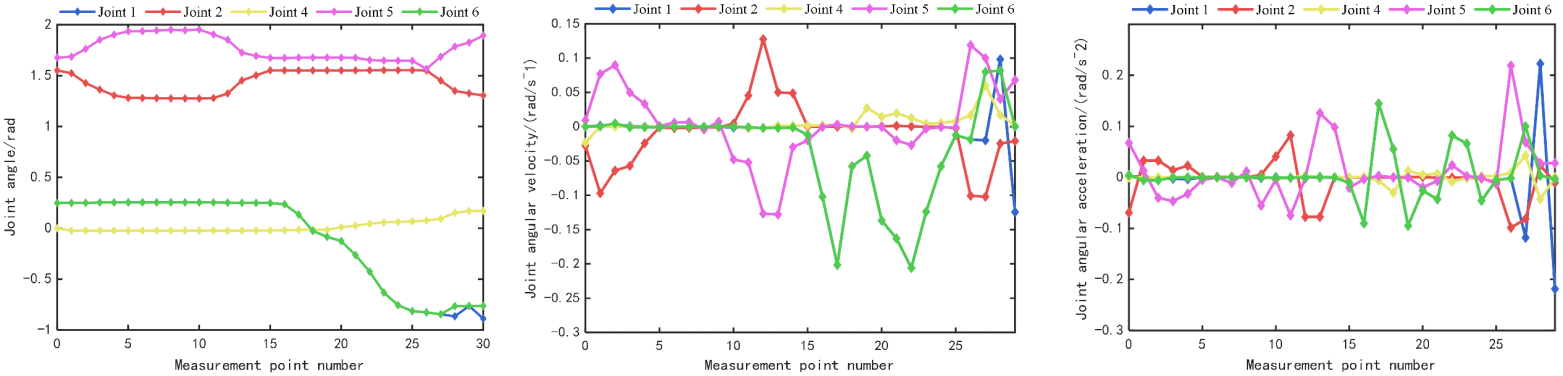
Actual parameter curves of rotational joint. **(a)** Actual angle of rotational joint; **(b)** Actual angular velocity of rotational joint; **(c)** Actual angular acceleration of rotational joint.

The above key points were recorded, and the data were imported into MATLAB to plot the approximate trajectory of the robotic arm, as shown in Fig 20. A total of 30 points were selected, including the aforementioned key points, which were labeled in sequence. The parameters of each joint were exported, and curves were plotted for comparison with the simulation curves, as shown in Fig 21.

Since the position of the translational joint 3 is relatively fixed, the actual parameter curve for it is not plotted separately. Analysis shows that the actual joint curves are highly similar to the simulation curves. However, there are cusps at the turning points and peak values, which is attributed to the insufficient number of selected points, affecting the smoothness of the curves.

Finally, RRT*, D-RRT, Bi-RRT*, A-RRT, and the AS-DTRRT algorithm proposed in this paper were used for path searching. All algorithms share identical initial nodes, target nodes, and workspace ranges. The initial step size is uniformly set to 100 mm, the maximum number of iterations is 10000, and the planning time threshold is 200 s. For the proposed AS-DTRRT algorithm, the distance sensitivity coefficient α is initialized to 0.8, and the target direction bias factor $\rho_{step}$ is initially set to the range (0,1]. The core parameters of the comparison algorithms are matched with those of the proposed algorithm to eliminate the influence of initial condition differences on the experimental results. When the number of iterations or the time threshold is exceeded, the search is deemed a failure, and the next search is performed immediately. All algorithms adopted pipeline obstacle-avoidance strategies and fifth-order polynomial interpolation for trajectory planning. Experiments were conducted under the same joint velocity conditions, with each algorithm tested 10 times. The average trajectory length, average operation time for complete trajectories, and successful operation counts were compared. The experimental results are shown in Table 3, where all data are rounded to integers for convenient measurement.

**Table 3:** Comparison of experimental results of different algorithms.

Search algorithms	Average time/s (μ ± σ)	Average Path/mm (μ ± σ)	Successful Trials
RRT*	178 ± 8.2	7967 ± 125	7
D-RRT	168 ± 7.5	7284 ± 108	8
Bi-RRT*	149 ± 6.1	6742 ± 92	8
A-RRT	170 ± 6.8	6920 ± 98	9
AS-DTRRT	142 ± 4.3	6280 ± 76	10

In Table 3, μ denotes the mean value and σ the standard deviation, indicating the fluctuation range of each search value. As shown in Table 3, AS-DTRRT outperforms the other four algorithms in all aspects. Specifically, in terms of actual operation time, it reduces by 20.22%, 15.48%, 4.69%, and 16.47% respectively compared to the four algorithms. In terms of average path length, it shortens by 21.17%, 13.78%, 6.85%, and 9.24% respectively. In terms of successful operation counts, AS-DTRRT achieves 100% success rate in 10 trials, demonstrating high stability. Compared with other algorithms, the standard deviation of time and path trajectories is smaller, indicating higher search stability.

Test results demonstrate that the AS-DTRRT algorithm optimizes sampling efficiency through target direction bias factors and simplifies collision detection processes by integrating pipeline obstacle avoidance strategies. While maintaining path planning accuracy, it significantly reduces both time complexity and computational load. These findings conclusively prove that the AS-DTRRT algorithm can operate stably and in real-time on automotive embedded platforms, fully meeting the real-time performance and reliability requirements for underground pipeline installation operations in coal mines.

## 5. Conclusions

(1) The pipeline installation robot installs pipelines on both sides of the mine roadway through the robotic arm. The pose of the robotic arm changes with the variation of pipeline position, and the geometric shape and length of the pipeline affect the movement trajectory of the robotic arm in a complex and changeable working environment. Aiming at the problems of long manipulator movement trajectory, low efficiency, and easy pipeline collision during pipeline installation, a trajectory planning method for pipeline installation robots based on the improved dual-tree RRT algorithm (AS-DTRRT) is proposed.
(2) By integrating the Artificial Potential Field method and introducing an adaptive step-size dynamic environment biasing strategy, the growth direction of new nodes is guided to enhance path search capability. The influence of pipeline geometry and length on the robotic arm workspace is analyzed, and a pipeline obstacle-avoidance strategy is proposed considering the operational conditions. Fifth-order polynomial interpolation is used to conduct trajectory planning for the collision-free path obtained from the search, meeting the requirements of operational scenarios.
(3) To verify the practical effectiveness of the AS-DTRRT algorithm and the rationality of trajectory planning, four algorithms, namely RRT*, D-RRT, Bi-RRT*, and A-RRT, were selected for comparison. In the actual operational conditions, AS-DTRRT demonstrates excellent comprehensive performance. Experimental results show that compared with the other four algorithms, AS-DTRRT reduces the actual operation time by 20.22%, 15.48%, 4.69%, and 16.47%, respectively, and shortens the average path length by 21.17%, 13.78%, 6.85%, and 9.24%, respectively. In terms of successful operation counts, it achieves 100% success rate in 10 trials, indicating high stability. The trajectory planning method based on AS-DTRRT can provide technical support for trajectory planning of similar coal mine robots.
